# Synthesis of an Enzyme‐Triggered Chitosan‐Based Drug Delivery System for Peri‐Implantitis Prevention

**DOI:** 10.1002/chem.202501800

**Published:** 2026-02-15

**Authors:** Nelly Senze Nnane, Amit Gaikwad, Muhammad Imran Rahim, Andreas Winkel, Oliver Hergert, Till Beuerle, Meike Stiesch, Henning Menzel

**Affiliations:** ^1^ Institute for Technical Chemistry TU Braunschweig Braunschweig Germany; ^2^ Department of Prosthetic Dentistry and Biomedical Materials Science Hannover Medical School Hannover Germany; ^3^ Institute of Transplant Immunology Hannover Medical School Hannover Germany; ^4^ Lower Saxony Centre For Biomedical Engineering Implant Research and Development Hannover Germany; ^5^ Institute for Pharmaceutical Biology TU Braunschweig Braunschweig Germany

**Keywords:** cleavage reaction, click chemistry, drug delivery, enzymes, polymer

## Abstract

Dental implants have become a leading solution for tooth loss, yet bacterial infections remain a major complication. Antibacterial implant coatings are an important approach to reduce or prevent bacterial infections at the implant. Given the oral cavity's complex microbiome and the importance of some commensal bacteria, it is crucial to develop antibacterial implant coatings that release the active ingredient only when needed. In this work, an enzyme‐triggered drug delivery coating based on chitosan with ciprofloxacin as antibiotic was synthesized. The ciprofloxacin is covalently bound via a self‐immolative linker with an enzyme‐labile group. The linker‐drug conjugate was bound to modified chitosan through a Diels–Alder click reaction. A stable drug‐delivering coating was assembled on titanium by alternating cationic chitosan with the drug with anionic alginate using layer‐by‐layer dip‐coating. Ellipsometry was used to characterize the coating and demonstrating its stability. The function of the enzyme‐labile self‐immolative linker was clearly demonstrated by release experiments and detection of genuine ciprofloxacin using UHPLC/HRAM‐MS and MS/MS. Furthermore, its biocompatibility was confirmed. After enzymatic activation, the coating exhibited antibacterial activity against an *A. naeslundii* biofilm. This provides proof of principle for this enzyme‐responsive approach to drug delivery.

## Graphical abstract

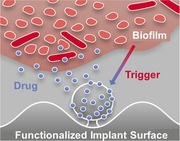

## 1 Introduction

Over the past years, the number of patients with dental implants has increased, as they are a superior alternative for patients with missing teeth, with a success rate of more than 89% [1]. Despite this mayor breakthrough in the dental restorative practice and the advantages these implants offer, the problem of implant failure due to biological causes remains a limitation [2]. Peri‐implant mucositis and peri‐implantitis are diseases caused by the penetration of bacteria into the area surrounding the implant. Whereas peri‐implant mucositis is a reversible infection of the soft tissue, peri‐implantitis results in irreversible damage to both the soft and the hard tissues around the implant and, in severe cases, leads to loss of the implant. Due to the formation of bacterial biofilms in which bacteria are less susceptible to antibiotics, antibacterial treatment options for peri‐implantitis are less effective [3].

Treatment options for this disease are limited and resource‐intensive, that is why prevention is a high priority in clinical practice. Among other prevention methods such as physical or mechanical modification of the implant surface, chemical modification such as the use of antiadhesive coatings, contact killing coatings or drug release coatings seem promising [4, 5, 6, 7, 8]. However, anti‐adhesive and contact‐killing antibacterial surfaces are limited by their inability to deal with bacteria within the biofilm that do not come into contact with the implant surface. Additionally, the effectiveness of such systems is often compromised by the accumulation of dead bacteria and associated debris on the surfaces. Passive drug delivery systems often result in premature and uncontrolled release of excessive amounts of antibacterial agents in the initial phase, which can lead to cytotoxicity in the surrounding tissue. In this context, the search for drug delivery systems (DDS) that release drugs in a controlled and selective manner, for example, in the presence of certain bacterial enzymes as triggers, is of interest.

Due to the overexpression of enzymes involved in pathological conditions, enzyme‐responsive DDS are an interesting approach in the field of infection and cancer therapies. Delivery systems with enzyme‐labile linkers between drug and a polymeric carrier enable the release of antibiotics on demand. For instance, Bourgat et al., prepared a DDS based on poly‐L‐lysine conjugated with ciprofloxacin and combined with alginate to form hydrogel nanoparticles [9]. The intended cleavage of the poly‐L‐lysine peptide linkage by trypsin and the release of ciprofloxacin was proven. Unfortunately, the chemical modification of the ciprofloxacin necessary for the conjugation, which remained at the drug after the enzymatic cleavage compromised the antibacterial effect significantly [9].

Self‐immolative linkers are defined as covalent groups between a carrier and a drug that can be cleaved by a stimulus such as an enzyme and subsequently decompose in a domino reaction. This results in the release of the original drug without residues of the linker [10]. The release of the desired molecules can be tailored to be specific and selective with using specific enzyme labile groups. This makes the DDS stable in the absence of the corresponding bacterial enzyme. Grether et al. reported on such an enzyme‐labile self‐immolative linker. The system was shown to be stable under different reaction conditions and released model compounds in a pure form in the presence of penicillin‐G‐amidase (PGA) [11].

Extending this concept and taking advantage of a biodegradable polymer, it would be interesting to couple such self‐immolating linkers to chitosan to create a DDS capable of installing antibiotics on the surface of implants and thus at the most likely location of a possible infection. Chitosan is of particular interest because it is biodegradable and biocompatible and has already been shown to be useful in the production of stable and cell‐compatible coatings on implants. In addition, chitosan has antimicrobial properties. Unlike other polymers such as hyaluronic acid or alginate, chitosan is a polycation and the cationic character plays a unique role in its interaction with titanium surfaces. In addition, it offers the possibility of producing multiple layers via layer‐by‐layer deposition. Previously, the use of chitosan‐based DDS to form coatings on Titanium implants has been reported [9, 12, 13, 14, 15, 16]. The use of chitosan for the delivery of fluoroquinolone antibiotics such as ciprofloxacin and moxifloxacin has already been reported [17]. For instance, Egorov et al. reported the synthesis of a ciprofloxacin‐chitosan DDS, in which ciprofloxacin is conjugated to the chitosan backbone through a pH‐sensitive hydrazone linker. At a pH of 6.5, ciprofloxacin was steadily released from the chitosan backbone [18]. Although infections are often associated with a decrease in pH in the surrounding tissue, a release based on a pH shift is less selective than an enzymatic reaction.

Both ciprofloxacin and moxifloxacin are FDA approved drugs used to treat a wide range of infections. Compared to ciprofloxacin, moxifloxacin would be the better choice for treating oral bacterial biofilm infections,[19, 20, 21] but because of its better availability, cost‐effectiveness, and good detectability ciprofloxacin was chosen as model compound for this class of antibiotics here. However, the same chemistry would also work with moxifloxacin.

Click reactions are particularly useful for the conjugation of a drug system to a polymer scaffold [22, 23]. These reactions take place under mild conditions, provide high yields and form no or only benign byproducts. In particular, the azide‐alkyne‐Huisgen 1,3‐dipolar cycloaddition and the thiol‐ene reaction are frequently used. Copper, which is used as a catalyst in these azide‐alkyne reactions, forms complexes with chitosan and is therefore difficult to remove [9]. The Diels–Alder cycloaddition is another click reaction that does not require any metal catalyst, hence it is well‐suited for biomedical applications [24], and has been proposed for chitosan derivatives [25].

This work describes the preparation of an enzyme‐triggered DDS. Key steps were functionalization of chitosan with 3‐maleimidopropanoic acid and coupling it with a furan‐functionalized self‐immolative linker‐drug conjugate employing a Diels–Alder reaction. The linker‐drug conjugate was prepared in a multistep synthesis. The DDS was used to form stable coatings on titanium surfaces by layer‐by‐layer deposition of the cationic chitosan derivative with anionic alginate. Furthermore, the stability, biocompatibility and antibacterial activity of the drug delivery coating were tested with and without penicillin‐G‐amidase.

## 2 Results and Discussion

**SCHEME 1:**
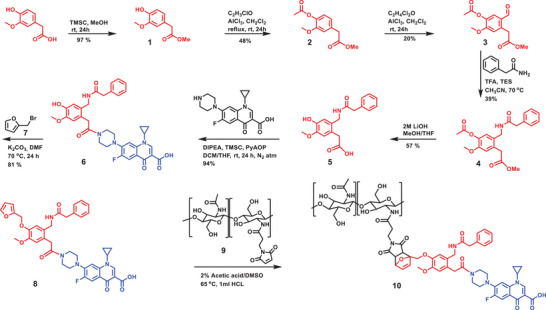
Synthesis of self‐immolative linker‐ciprofloxacin‐system.

**FIGURE 1:**
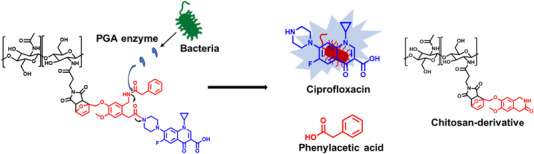
Domino reactions releasing pure ciprofloxacin (blue), which can kill bacteria, from the polymer support (black), using penicillin‐G‐amidase (PGA) as a trigger for the self‐immolative linker (red).

The DDS was prepared by combination of a chitosan‐maleimide derivative with a furan‐functionalized self‐immolative linker‐drug conjugate, prepared in 8 synthetic steps, as shown in Scheme 1. Briefly, a self‐immolative linker containing a PGA‐labile group (**5**) bound to ciprofloxacin (**6**) was prepared in 6 synthetic steps. Compound **6** was then reacted with a furan derivative (**8**), which was subsequently reacted with the dienophile of the chitosan‐maleimide derivative in a Diels–Alder reaction to yield compound **10**. The linker incorporates a phenylacetamide group, which can be recognized and selectively cleaved by PGA, and the desired drug (ciprofloxacin) can be released in a pure form by a domino reaction (Figure 1).

The central element of the release system is the enzymatically cleavable and self‐immolative linker (**5** in Scheme 1). This was synthesized starting from homovanillic acid, including a two‐step protection by esterification reactions at the carboxylic acid and the phenolic OH group using alcohol and acid chloride, respectively. The yield of the intermediates **1** and **2** was 97% and 48%, respectively [26, 27]. The use of AlCl_3_ in the esterification reaction was beneficial and the resulting product could be purified without difficulty for the next reaction, which was the introduction of an aldehyde by an acylation reaction (**3**). The structure was characterized by ^1^H‐NMR analysis and characteristic peaks were observed at 3.62 and 2.24 ppm, respectively (see Supporting Information).

In the synthetic route published by Grether et al., the introduction of the phenylacetamide group is accomplished by coupling with N‐(hydroxymethyl)‐2‐phenylacetamide in a mixture of acetic acid and sulfuric acid. However, the small variation in the target structure in our synthesis route—ester instead of an ether group at position 4,—led to severe side reactions and the formation of tar‐like products when using this acetylation method. Therefore, a milder variant was used for the acetylation reaction. First, an aldehyde was introduced to produce **3**. This was then reacted with 2‐phenylacetamide in a reductive N‐alkylation to give **4** in 39% yield [28]. Subsequent, through saponification reaction with LiOH [29], **4** was selectively deprotected back to the acid and OH─ functionalities **5** in 58% yield, required for binding the antibiotic and to the polymer backbone (see Supporting Information).

As mentioned above ciprofloxacin was selected as a model antibiotic for this study due to its availability, cost‐effectiveness and detectability. To bind the ciprofloxacin, its secondary amino group was reacted with the acid functionality of **5**, employing an amidation reaction yielding **6** (94%) (see Supporting Information) [9].

The furan‐functionalized linker can be introduced by reacting furfuryl bromide or furfuryl alcohol with compound **6** by Williamson etherification or Mitsunobo reaction, respectively. The Mitsunobu reaction is straightforward but has a lower yield, and the purification process is complex due to the many reagents involved in the reaction. Therefore, using furfuryl bromide in a Williamson etherification was a suitable alternative. Furfuryl bromide, was prepared from furfuryl alcohol by bromination, and it was immediately reacted with **6** to give **7** in 81% yield. The structure was characterized by ^1^H‐NMR analysis showing characteristic furan peaks at 6.57–6.48 and 5.19 ppm (see Supporting Information).

**FIGURE 2:**
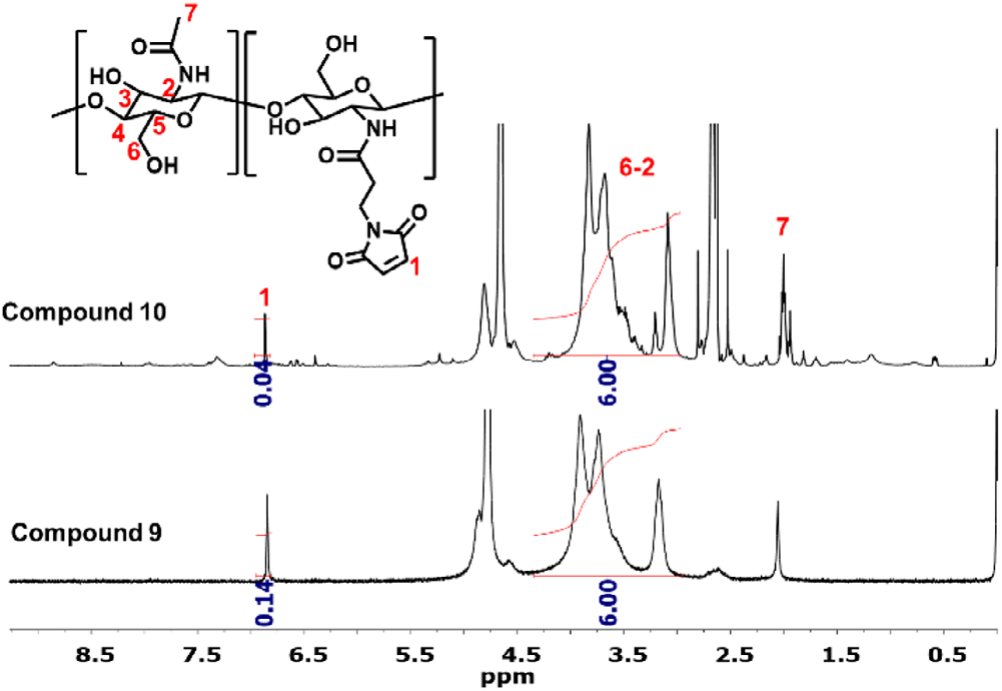
1H‐NMR spectra showing the change in the intensity of maleimide proton after Diels–Alder reaction to form compound **10**.

Since the drug delivery system is intended for the modification of implant surfaces as release coating, the use of a polymer support is necessary. Chitosan was chosen since it is already well‐established in many biomedical applications, such as tissue engineering and DDS [9, 15, 30, 31, 32, 33]. Also, it is inexpensive and readily available in medical grade. For this investigation, chitosan obtained from shrimp shells was used; however, it was not yet medical grade. Therefore, further purification was carried out by heating chitosan in a NaOH solution, dissolved in slightly acidic water and dialyzed against a 0.1 M NaCl solution to remove any protein residues and other impurities [33, 34]. The purified chitosan with a molecular weight of 640,000 g/mol and a degree of deacetylation of about 90% (amount of free amine groups) as determined by GPC and ^1^H‐NMR, respectively, was water soluble at slightly acidic pH conditions. The chitosan was further modified with maleimido propionic acid according to Matsumoto et al. [35] yielding **9**. The structure was confirmed by ^1^H‐NMR analysis, showing a corresponding signal at 6.85 ppm which can be attributed to the H─C═C─H protons of the maleimide (see Supporting Information). The ^1^H‐NMR spectra show that a degree of substitution of 6.5% has been achieved. The presence of maleimide improved solubility in aqueous media and allowed the binding of a furan‐based linker system **8** through Diels–Alder reaction to form **10**. This reaction involves the conjugation of the maleimide from **9** as dienophile and the furan of linker **8** as diene by cycloaddition at 65°C. From the integration of the maleimide protons at 6.85 ppm relative to the peaks corresponding to the chitosan backbone at 4.4–3.0 ppm, the amount of maleimide on the backbone of chitosan (DS) before and after Diels–Alder reaction was calculated, according to equation 1. Where I(1) represent the integral of the maleimide peak, I(6‐2) is the integral of chitosan peak, 6 is the number of protons on chitosan repeating unit, and 2 is the number of protons of maleimide. The difference in maleimide units after Diels–Alder reaction is a result of the conjugation of furan‐linker to the chitosan‐maleimide backbone and indicated a degree of substitution of 4.5% with the drug‐linker‐conjugate (see Figure 2).

(1) $$DS\;\left[\%\right]\;=\;\frac{\frac{1}{2}I\left(1\right)}{\frac{1}{6}I\left(6-2\right)\;+\;\frac{1}{2}I\left(1\right)}$$

**FIGURE 3:**
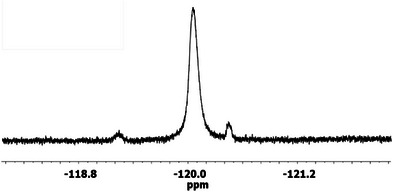
19F‐NMR spectra of the chitosan‐bound linker showing the characteristic peaks of ciprofloxacin.

**FIGURE 4:**
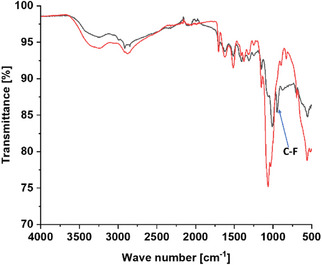
FTIR spectra showing the signals of chitosan‐maleimide (red) and chitosan‐bound linker DSS (black).

Furthermore, in the ^19^F‐NMR spectrum of the final product (Figure 3), the fluorine signal of ciprofloxacin was observed at −120.05 ppm, surrounded by asymmetrical spinning satellite signals at −119.23 and −120.45 ppm. These latter signals arise from the interaction between the ^19^F nuclei and nearby carbon nuclei, highlighting the high sensitivity of the ^19^F nucleus (more sensitive than the ^13^C nucleus) not only to internal effects but also to distant interactions [36]. Additionally, the conjugation of the immolative linker to the chitosan‐maleimide (CS‐Ml) backbone was confirmed by the appearance of the C─F vibration at approximately 949 cm^−1^ in the IR spectrum. However, in ^1^H‐NMR spectrum, all peaks attributable to the linker were buried under the broad chitosan peaks (Figure 4). This was expected, as the degree of substitution is only 4.5%, hence these signals are relatively low compared to those of chitosan, the main component.

Finally, the chitosan‐bound linker system **10** was dissolved in a 2% acetic acid /DMSO mixture (pH of 2.37) and purified alginate was dissolved in water (pH of 6.72). The concentration in both cases was 4 mg/mL. These solutions were used to produce coatings on Ti‐plates in a layer‐by‐layer (LbL) dip‐coating process. This LbL‐technique is based on the electrostatic interaction of positive and negative‐charged polyelectrolytes. Before the coating process the Ti‐plates were cleaned in an air plasma to remove all residues on the surface and promoting a better adhesion of coatings. The negatively charged Ti‐plates were dipped in a solution of the positively charged chitosan‐based DDS **10** for about 10 min. The plates were washed with distilled water and dried with a stream of N_2_. Subsequently, the plates were dipped into an alginate (negatively charged) solution for 10 min, washed with distilled water and dried with a N_2_‐stream [31, 37]. This process was repeated 10 times to form alternating layers while showing sufficient deposition of the antibiotic compound on the surface.

**FIGURE 5:**
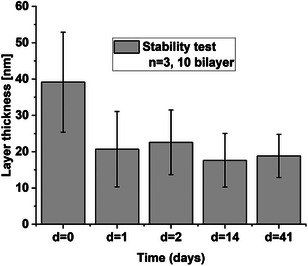
Dry thickness of a 10‐layer chitosan with linker and drug / alginate before and after longterm immersion in phosphate buffered saline at pH 7.4 for 1, 2, 14, and 41 days, (*n* = 3).

The dry layer thickness of the coating after 10 LbL‐cycles was determined by laser ellipsometry to be 39 nm ± 13.8 for a set of three samples (*n* = 3). The stability of the coating was tested by long term immersion in phosphate buffered saline at pH 7.4 (37°C). A decrease in layer thickness from 39 nm ± 13.8 to 22 nm ± 8.9 was observed after 1 day as shown in Figure 5. This decrease after 1 day could be due to a wash‐out of residual polymer. However, after 2, 14, and 41 days, no difference in layer thickness was noticed, indicating the stability of the coating. This stability test shows that this system can be used in an oral bacterial biofilm model [38], which will be used to test the biocompatibility and drug release potential.

Furthermore, the coatings were also tested for the release of ciprofloxacin under the influence of PGA. The coated Ti‐plates were prepared as described above using LbL and then incubated with a buffer solution containing either PGA, no PGA, or denatured PGA. By comparison with authentic ciprofloxacin enzymatically released ciprofloxacin was unequivocally detected in the filtrate using UHPLC‐MS and MS/MS analysis. This is a clear indication that the enzyme‐labile self‐immolative linker is functioning. The signal is very strong in the presence of PGA, while only a very small amount of ciprofloxacin can be detected without active PGA (see Figure S21).

### 2.1 Biological Testing

To confirm the suitability of the DDS for biomedical applications, antibacterial activity and cytocompatibility were systematically evaluated considering the functionality by enzymatic cleavage for the intermediate compound **6** as well as for a coating prepared from the chitosan‐drug‐conjugate **10** and alginate.

#### 2.1.1 Antibacterial Activity of Compound **6** Against *Actinomyces naeslundii*

**FIGURE 6:**
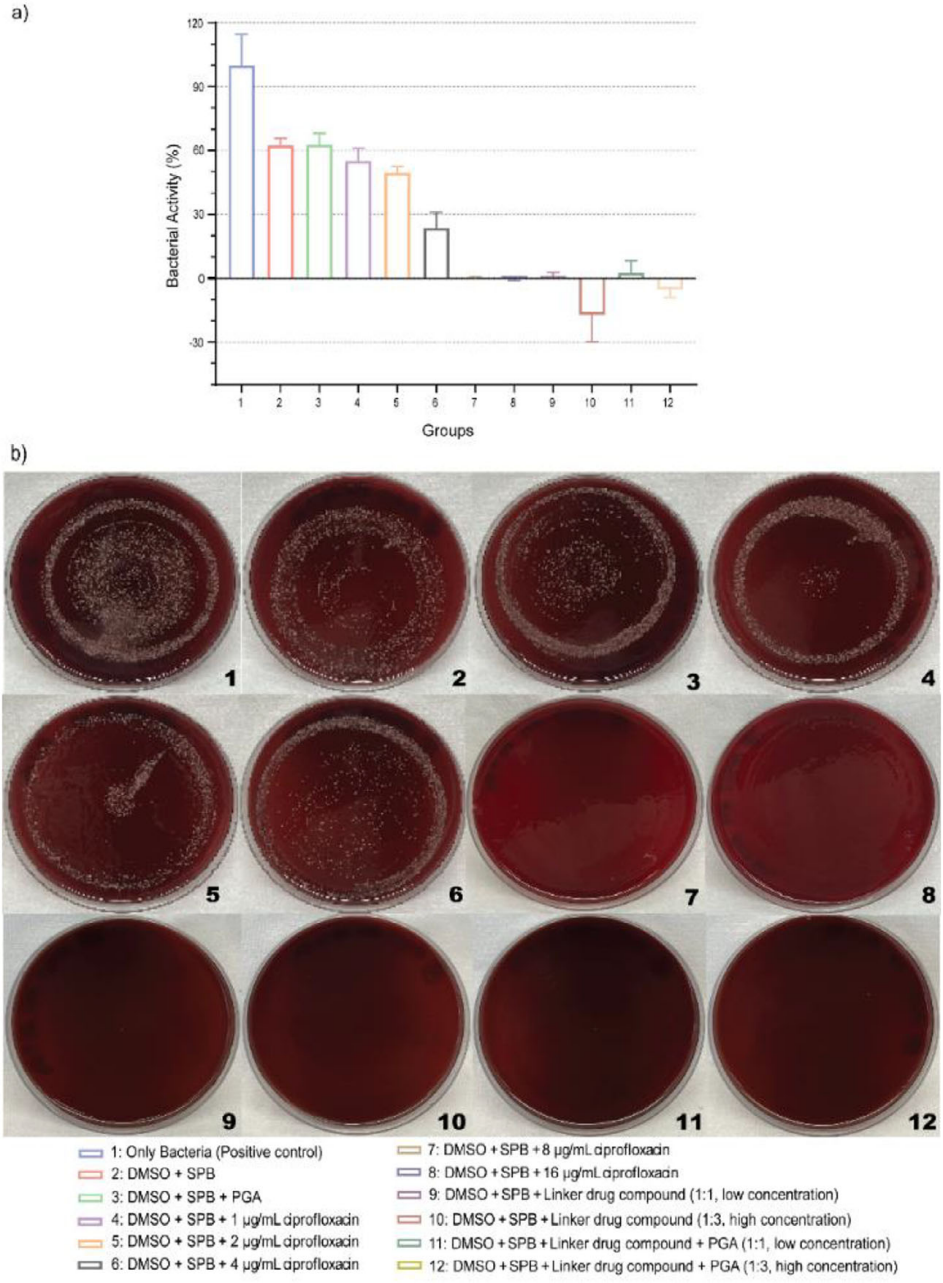
Antibacterial activity of linker‐drug compound **6** after 24 h incubation against *A. naeslundii*. (a) Percentage of bacterial growth activity assessed using broth dilution assay. (b) CFUs analysis on FAA plates after 48 h incubation.

To evaluate the release of functional ciprofloxacin after cleavage of compound **6** using PGA, antibacterial activity assessments were performed against *A. naeslundii* a pathogen associated with peri‐implant diseases [39]. The growth of planktonic bacteria in the positive control (group 1) measured by broth dilution assay after 24 h of incubation was considered to represent the strongest bacterial activity and thus set as 100%. In comparison, the solvent alone (DMSO + PBS, group 2) caused in average 40% reduction in bacterial activity (see Figure 6a), which however was nonsignificant (*p* > 0.01). The additional application of PGA to bacteria demonstrated no changes in this effect. However, spreading the bacteria on culture plates did not show a corresponding reduction in the amount of colony forming units (CFU) in either case, which suggests that these observations are more related to a growth‐inhibiting rather than a killing effect of the solvent (see Figure 6b). In contrast, the addition of ciprofloxacin in increasing concentrations (groups 4, 5, 6, 7, 8) led to a further decrease in bacterial activity, reaching significance for the first time at 4 µg/ml (*p* < 0.05), before bacterial growth was no longer detectable at 8 µg/ml (*p* < 0.0001), which was confirmed by CFU analysis (see Figure 6a,b). Therefore, the treatment of *A. naeslundii* with ciprofloxacin concentrations of 8 µg/ml and higher was proven to be sufficient to prevent any bacterial growth under these conditions. For the linker‐drug conjugate **6**, neither in the broth dilution assay nor in the CFU analysis, any bacterial growth of *A. naeslundii* could be detected for the investigated concentrations with or without enzymatic supplementation (groups 9, 10, 11, 12; *p* < 0.0001). These findings suggest a strong antibacterial effect by the linker‐drug conjugate **6** itself, even without enzymatic activation. This effect is well known for a wide range of nonapproved FDA ciprofloxacin derivatives formed by reaction of secondary amine functionality, which shows equal and/or superior activity profile than the parent ciprofloxacin [9, 40, 41] The bacteria activity profile of fluoroquinolones depends on the steric and electronic environment of the substituent at C‐7 group, which influences the interaction with the targeted bacteria, as has been shown for fourth generation Delafloxacin derivatives [42]. Thus, the functional release of ciprofloxacin by enzymatic activity cannot be fully clarified from the experiments and needs further exploration.

#### 2.1.2 Cytocompatibility of Compound **6** According to Cell Membrane Integrity and Metabolic Activity of HGFs

**FIGURE 7:**
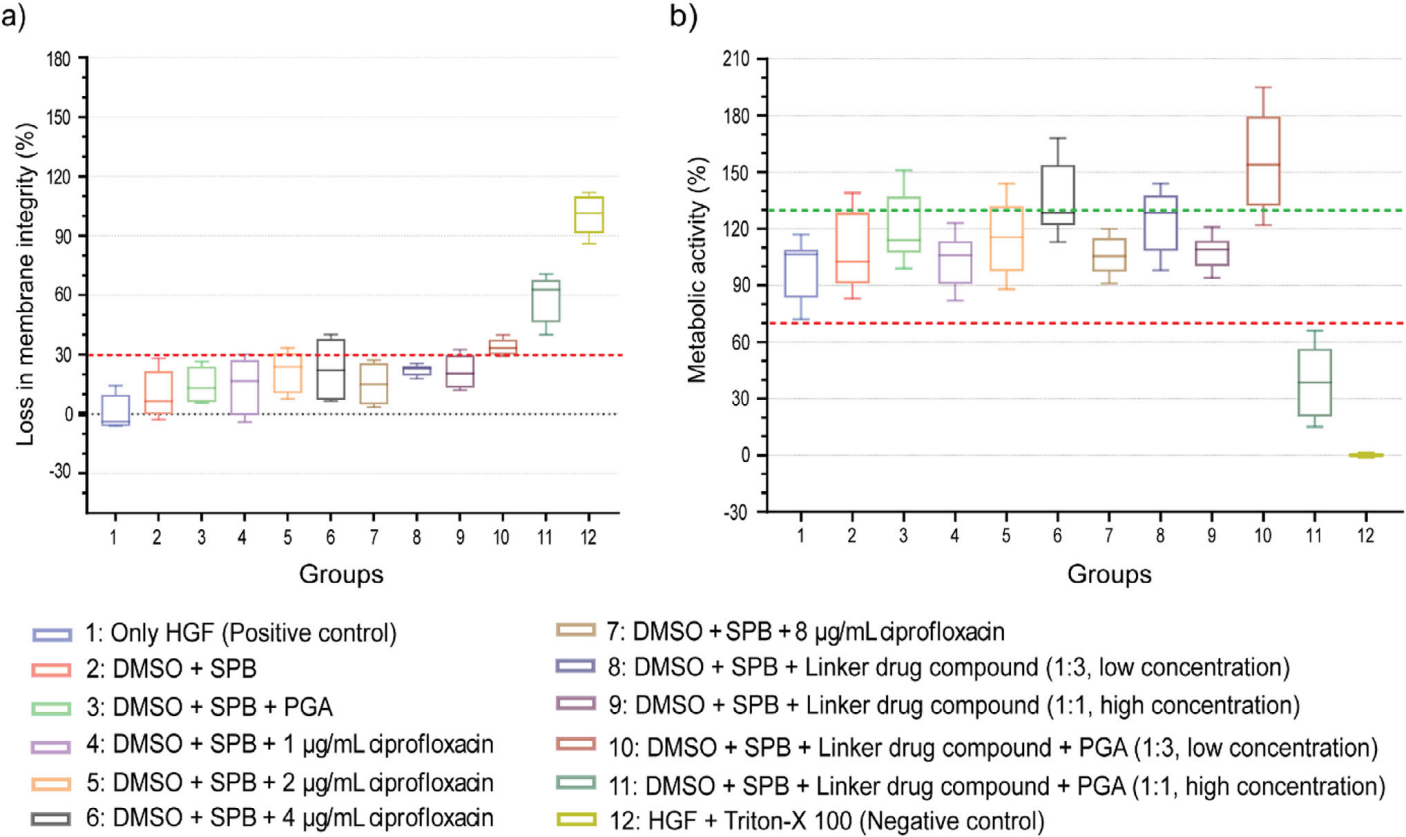
Cytocompatibility of linker‐drug compound **6** after 24 h incubation with HGFs. (a) Cell membrane integrity estimated by measuring the extracellular LDH activity within supernatants. (b) Percentage of cell metabolic activity assessed using CellTiter‐Blue Cell Viability Assay.

Cytocompatibility assays were performed to explore further the working principle of linker‐drug conjugate **6** and their products in presence of Human Gingival Fibroblasts (HGF). To determine the effects of compound **6** on membrane integrity, the activity of extracellular LDH in the supernatant of HGFs was normalized compared to the untreated positive control (0%, group 1) and the negative control treated with Triton‐X 100 (100%, group 12). LDH activities above 30% were considered cytotoxic (red dotted line, see Figure 7a). The addition of solvent alone (DMSO + PBS, group 2), and in combination with PGA (group 3) or different concentrations of ciprofloxacin (groups 4 to 7) demonstrated only moderate, nonsignificant release of LDH below the 30% threshold (*p* > 0.01). Even the addition of the linker‐drug conjugate **6** at different concentrations (groups 8, 9) did not lead to any indication of cytotoxicity. This indicates a general cytocompatibility of the investigated compound solubilized in DMSO + PBS. However, in presence of PGA compound **6** caused significant increases in extracellular LDH activity (34%, group 10; 62%, group 11; *p* < 0.01) indicating PGA‐mediated cleavage and release of ciprofloxacin.

These concentration‐ and enzyme‐related effects were confirmed measuring metabolic activity in HGFs (see Figure 7b). In comparison to the positive control (100%, group 1), the addition of solvent alone as well as in combination with PGA, different concentrations of ciprofloxacin or the linker‐drug conjugate **6** alone (groups 2 to 9) caused only moderate increases – in average not exceeding 130% (green‐dotted line), which would indicate severe cell stress. In contrast, the combination of lower concentrations of linker‐drug conjugate **6** with PGA led to a significant higher stress‐related metabolic activity (155%, group 10; *p* < 0.0001). For high amounts of compound **6** in combination with PGA even a distinct decrease in metabolic activity below the 70% threshold (marks progressive cell death) could be observed (40%, group 11; *p* < 0.0001).

Initiated by the trigger PGA, which can be produced by bacteria, hydrolysis at the amide linkage of compound **6** was expected to occur. In consequence, the self‐immolative linker released genuine ciprofloxacin accompanied by phenylacetic acid (PAA) and a dihydroisoquinolinone derivative, which however will remain bound in case of a chitosan‐based coating. The presented results highlight enzyme‐related changes within the soluble compound **6**, which in concentrations much higher than in any anticipated coating strategy resulted in adverse effects detectable according to metabolic activity and membrane integrity of human gingival fibroblasts. Unfortunately, it could not be conclusively clarified which of the mentioned substances were ultimately responsible, alone or in combination, for the observed cellular effects. However, ciprofloxacin as an antibiotic and phenyl acetic acid as an antibacterial agent and plant hormone with application in hyperammonemia and urea cycle disorders are therapeutics with a known spectrum of activity [43, 44]. For instance, PAA has been reported to show strong antibacterial activity against vaginal pathogens [45]. and has been proposed as a treatment for root cancer [44]. However, the results unequivocally show that the drug‐linker conjugate is cleaved by the enzyme.

#### 2.1.3 Antibacterial Activity of Chitosan‐Bound Linker DDS Coated on Titanium Plates Against *A. naeslundii* Biofilm

In the proposed DDS coating strategy with an LbL film of chitosan‐bound linker system **10** and alginate, significantly less ciprofloxacin is used in comparison to the investigations with the soluble compound **6**, as the layer thickness currently barely exceeds 40 nm. However, this is part of the concept, as the effect is to be exerted exclusively and directly on the surface to eliminate bacteria adhering there. The functionality and effectiveness of the coatings was tested using *A. naeslundii* and soft tissue cells in adhesion protocols.

**FIGURE 8:**
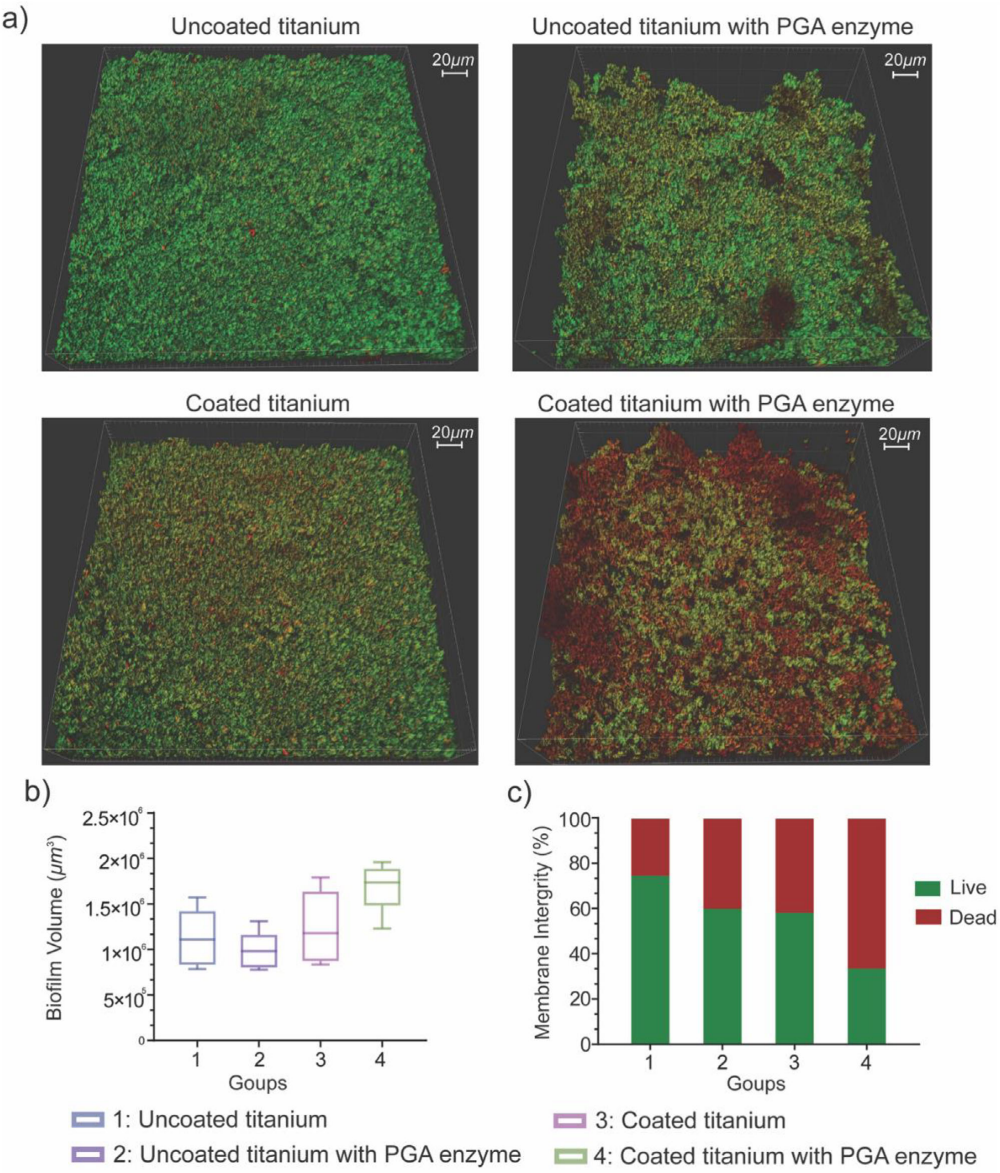
Antibacterial activity of chitosan‐bound linker DDS coated on titanium plates against *A. naeslundii* biofilm. (a) Representative 3D CLSM images showing a ‘green’ signal suggesting an intact bacterial cell membrane (live) and a ‘red’ signal highlighting cells with damaged bacterial membrane (dead). (b) Box‐plot diagram showing total bacterial biofilm volume after 48 h of incubation. (c) Bar diagram showing the distribution of live and dead proportions among the tested groups.

For antibacterial testing of chitosan‐bound linker DDS coating, *A. naeslundii* biofilms cultivated for 48 h on surfaces were analyzed. Bacterial biofilm on uncoated Ti‐plates showed strong green fluorescence after life‐dead staining, representing a robust biofilm formation (1.1 × 10^6^ µm^3^) with a 75% content of living bacteria (group 1, see Figure 8). Although the addition of PGA or usage of coated specimens alone ultimately resulted in more membrane permeability (proportion of living bacteria appeared reduced in both cases to 60%), no major changes in biofilm volume could be observed (groups 2, 3; see Figure 8). In contrast, triggering coated titanium plates with PGA caused a decrease in bacterial viability to 30% and increased biofilm volume by more than 50% to 1.8 × 10^6^ µm^3^ (group 4, see Figure 8).

The findings suggest that the chitosan‐bound linker DDS alone facilitated some degree of antibacterial activity. Although it did not drastically influence biofilm formation, viability within the biofilm appeared reduced. This reduction was attributed to the inherent antimicrobial properties of chitosan against *A. naeslundii*, as demonstrated in a previous study [46, 47]. Whether PGA has similar properties has not yet been investigated. The enzymatic activity on coated titanium plates though resulted not only in a rise in dead bacteria but was accompanied with a notable increase in biofilm volume. Beside the antibacterial property of released ciprofloxacin as an antibiotic, these observations could be an indication for the disruption of biofilm matrices, which makes the biofilm more flocculent and loosely organized, leading to an expanded structure and greater apparent volume as well as less protected bacteria. This phenomenon has been documented in studies exploring biofilm dynamics and the effects of various treatments on biofilm structure [48, 49], and highlights the antimicrobial action of ciprofloxacin and its ability to penetrate and disrupt biofilms when released from DDS [50]. Furthermore, it suggests that the targeted enzymatic cleavage of the chitosan‐bound linker DDS at the amide linker released pure ciprofloxacin in sufficient concentrations to effectively reduce bacterial viability within a biofilm directly on the surface.

#### 2.1.4 Cytocompatibility Testing of Chitosan‐Bound Linker DDS Coated on Titanium Plates

**FIGURE 9:**
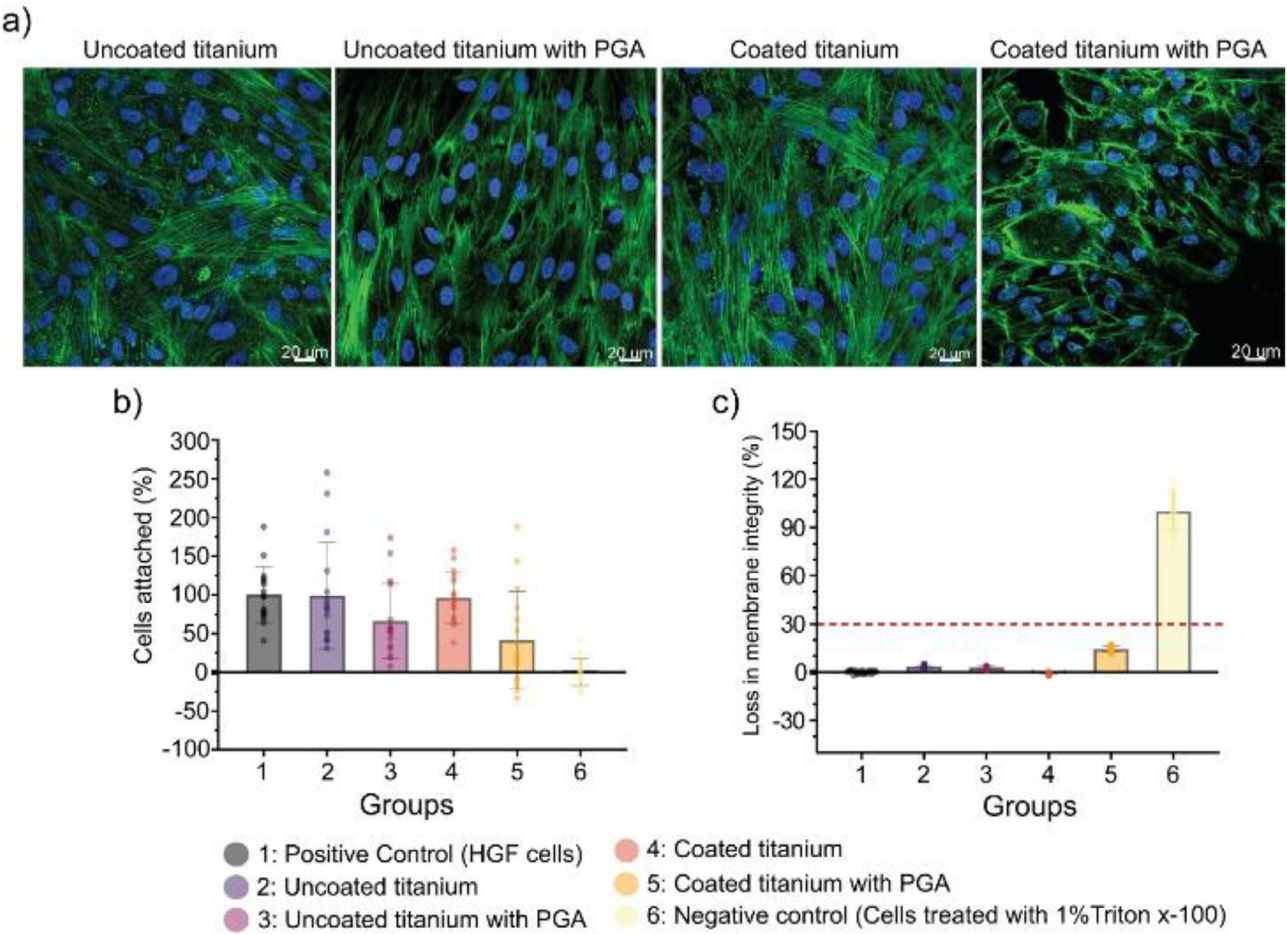
Cytocompatibility of chitosan‐bound linker DDS coated on titanium plates with and without supplementation of PGA after 48 h incubation with HGFs. (a) CLSM images showing HGF cell morphology with green cytoskeletal organization of actin filaments and round cell nuclei. (b) Amount of HGF cell adhesion on titanium plates. (c) Cell membrane integrity estimated according to LDH activity in supernatants.

For cytocompatibility testing and to monitor cellular adhesion on chitosan‐bound linker DDS coated surfaces, the growth of HGFs on titanium plates was assessed by fluorescence staining using confocal laser microscopy (CLSM). After 48 h of incubation, the HGF cell morphology on uncoated titanium plates displayed adequate cell attachment with well‐defined actin filaments and prominent cell nuclei (see Figure 9a).

The addition of PGA enzyme to the uncoated titanium did not alter the HGF cell morphology and the observed reduction in attached cells was not statistically significant (group 3; *p* > 0.05; see Figure 9a,b). Furthermore, membrane integrity of cells appeared unaffected (see Figure 9c). The applied coating alone also did not significantly alter the quantity, morphology or membrane integrity of the adherent human gingival fibroblasts (group 4; *p* > 0.05; see Figure 9a–c). Notably, the cells on this coated titanium even displayed elongated cytoskeletal organization forming a strong inter‐cellular network. When supplemented with PGA, HGFs on coated titanium showed alterations at certain locations with more round morphology and less pronounced actin filaments, indicating disruption in cytoskeletal organization (see Figure 9a). These effects appeared not uniform across the surface, but led overall to a significantly reduced membrane integrity (still well below the threshold of 30% in comparison to the control; group 5; *p* < 0.0001) as well as less attached cells (nonsignificantly; group 5; *p* > 0.05; see Figure 9b,c).

Above findings indicate that the chitosan‐bound linker DDS demonstrated optimal cytocompatibility by supporting cell attachment, facilitating cell spreading, and showing no cytotoxicity towards HGFs, consistent with previous literature attributing chitosan's cytocompatibility to its structural similarity with glycosaminoglycans in extracellular matrices [51, 52]. Alginate, as the other component in the coating, is a biocompatible biopolymer widely used in tissue engineering for its hydrogel‐forming ability, therefore mimicking extracellular environments [53, 54]. When activated by PGA, cells attached to the coating demonstrated altered morphology and signs of attachment and membrane integrity loss, which could be associated with either changes in the surface characteristic or released substances like ciprofloxacin and phenyl acetic acid. The latter would be consistent with our observations on the enzymatic cleavage of the soluble compound **6**, if we assume an increased concentration of the active ingredients directly at the surface. However, the observed effects, probably due to products of the enzymatic cleavage, are limited and cell compatibility is maintained.

## 3 Conclusion

A coating for titanium implants was developed that releases an antibiotic drug triggered by the presence of Penicilin‐G‐amidase, a bacterial enzyme. The coating, which is applied to titanium by a layer‐by‐layer (LBL) dipping process using alginate as a polyanion, is based on a cationic chitosan polymer conjugated to ciprofloxacin via a self‐immolative linker. This self‐immolative linker was produced in good yields in a seven‐step synthesis, and conjugated to ciprofloxacin. The conjugate was then attached to the chitosan via a Diels–Alder reaction. The LBL coatings are sufficiently stable in phosphate buffered saline, mimicing physiological conditions. Incubation with and without the PGA enzyme demonstrated genuine ciprofloxacin from the coatings, as verified by UHPLC/HRAM‐MS and MS/MS. The system has proven to be cytocompatible with human gingival fibroblasts and showed an antibacterial effect upon enzymatic activation, effectively targeting *A. naeslundii* biofilms. Taken together, the in vitro experiments indicated that the targeted enzymatic cleavage of the chitosan‐bound linker released ciprofloxacin in sufficiently high concentrations to effectively reduce bacterial viability within a biofilm directly on the titanium surface. The layer‐by‐layer coating approach on titanium plates further confirmed the stability and functionality of the system, highlighting its potential as a responsive antibacterial strategy for dental implants. While the proof‐of‐concept has been established, future research should focus on optimizing the drug‐loading capacity to enhance antibacterial efficacy, particularly against a broader spectrum of oral pathogens. This innovative approach holds significant promise for addressing peri‐implantitis and improving the longevity and success of dental implants.

## Conflicts of Interest

The authors declare no conflicts of interest.

## Supporting information



The authors have cited additional references within the Supporting Information [1–13].

## Data Availability

The data that support the findings of this study are available from the corresponding author upon reasonable request.
