# Supplementary material for: Synthesis of an Enzyme‐Triggered Chitosan‐Based Drug Delivery System for Peri‐Implantitis Prevention
Source: Chemistry. 2026 Feb 15;32(16):e01800. doi: 10.1002/chem.202501800 (PMC13109680; doi:10.1002/chem.202501800)
Supplement: Supplementary file 1 — The authors have cited additional references within the Supporting Information [1–13]. [file CHEM-32-e01800-s001.pdf]

Synthesis of an enzyme-triggered Chitosan-based drug delivery system for peri-implantitis prevention

Nelly Senze Nnane[a], Amit Gaikwad[b,c], Muhammad Imran Rahim[b], Andreas Winkel[b], Oliver Hergert[a], Till Beuerle[c], Meike Stiesch[b], Henning Menzel[a]

a N.S. Nnane, O. Hergert, Prof. Dr. H. Menzel*
Institut for Technical Chemistry, TU Braunschweig
Hagenring 30, 38106 Braunschweig, Germany
E-mail: h.menzel@tu-braunschweig.de

b A. Gaikwad, Dr. M.I. Rahim, Dr. A. Winkel, Prof. Dr. M. Stiesch
Department of Prosthetic Dentistry and Biomedical Materials Science, Hannover Medical School, Hannover, Germany
Institute of Transplant Immunology, Hannover Medical School, Hannover, Germany
Carl-Neuberg-Str.1,30625 Hannover, Germany
and
Lower Saxony Centre for Biomedical Engineering, Implant Research and Development,
Stadtfelddamm 34, 30625 Hannover, Germany

c Dr. T. Beuerle
Institute for Pharmaceutical Biology, TU Braunschweig
Mendelssohnstrasse 1, 38106 Braunschweig, Germany

1 Materials and Methods

Medium molecular weight Chitosan (CS) with a degree of deacetylation (DDA) = 90 %, Mw = 190 000–310 000 g mol−1, 1-ethyl-(3-dimethylaminopropyl carbodiimide) hydrochloride (EDC), and hydroxy-benzotriazole monohydrate (HOBt), sodium alginate, ciprofloxacin (98%), triethylsilane (99%), trifloroacetic acid (99%), lithium hydroxide, Dichloromethyl methyl ether (98%), Trimethylsilychloride (TMSC, 99%), N, N-Diisopropylethylamine (99%), phosphorus tribromide (99%) were purchased from Sigma Aldrich (St. Louis, MO, USA). Homovanillic acid (98%), aluminum chloride (99%), acetyl chloride (98%) were purchased from ABCR (Karlsruhe, Germany). 2-phenylacetamide (98%) was purchased from TCI Ltd. (Tokyo, Japan), 7-Azabenzotriazol-1-yloxy)tripyrrolidinophosphonium hexafluoro-phosphate (PyAOP, 98%) was purchased from carbolution chemicals GmbH (St. Ingbert, Germany), 3-Maleimidopropionic acid (Mal) was purchased from BIOSYNTH s.r.o (Bratislava, Slovakia). All chitosan-based samples dialyzed using dialysis tube with MWCO = 14 KDa purchased from Carl Roth GmbH + Co. KG (Karlsruhe, Germany). All NMR solvents were obtained from Deutero GmbH (Kastellaun, Germany).

Phosphate-buffered saline at pH 7.4 was prepared by dissolving 1 tablet of phosphate saline purchased from sigma Aldrich (St. Louis, MO, USA) into 1L of distilled water.

1.1 Synthesis of Methylhomovanillate (**1**):[1]

To a suspension of homovanillic acid (30 g, 164,68 mmol, 1 eq.) in TMSCl (41.80 mL, 329.35 mmol, 2 eq), excess MeOH (480 mL) was added under stirring. After 48 h, all volatile components were removed under vacuum. Purification of the crude product was done using column chromatography in an ethyl acetate: hexane mixture (2:3). The solvent was evaporated by to get a white solid **1** (60.2 g, 318,04 mmol, 97%)

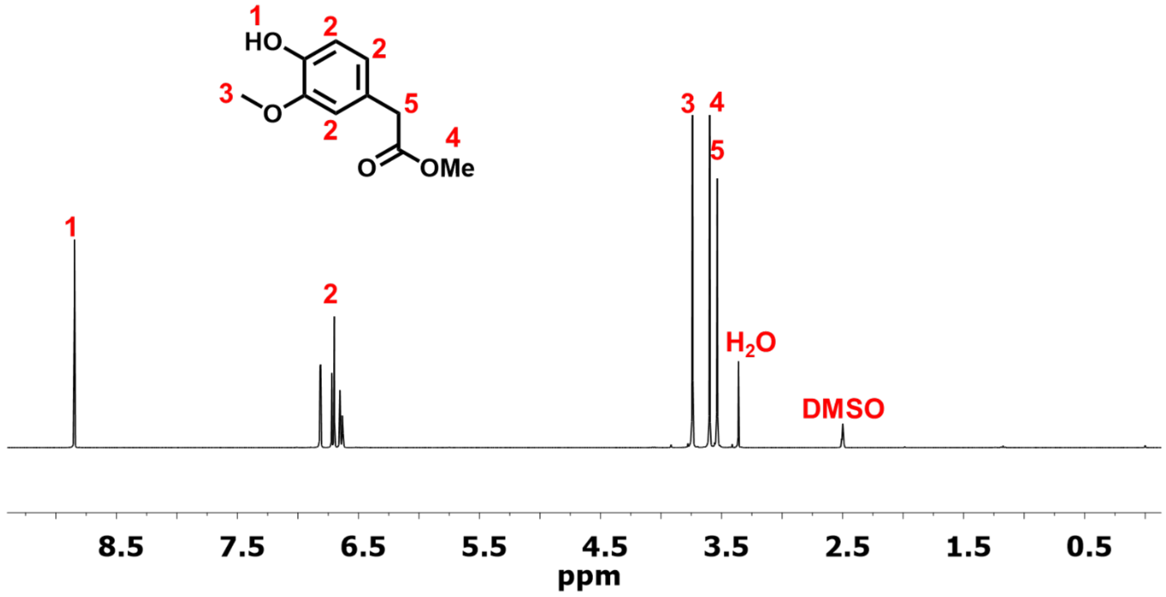

Figure S 1: ^{1}H-NMR (DMSO-d, 400MHz): δ [ppm] = 8.89 (s,1H, 1), 6.818-6.698 (m, 3H, 2), 3.77 (s, 3H, 3), 3.619 (s, 3H, 4), 3.537(s, 2H, 6)

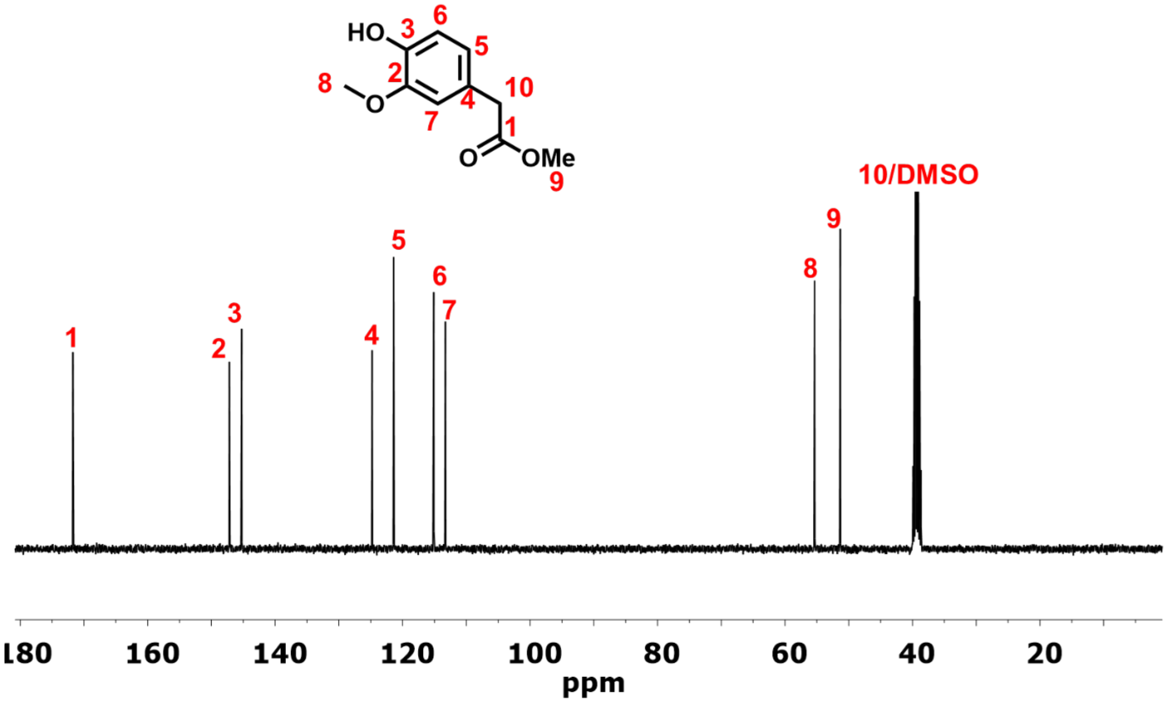

*Figure S 2: ^{13}**C-NMR (DMSO-d, 400MHz):** δ [ppm] = 171.74, 1), 147.18 (2), 145.25 (3), 124.78 (4), 121.40 (5), 115.12 (6), 113.29 (7), 55.36 (8), 51.33 (9), 40 (10)*

1.2 Methyl 2-(4-acetoxy-3-methoxyphenyl)acetate (**2**):[2]

In a 100 mL 3-neck round bottom flask equipped with a magnetic stirrer, and a reflux condenser with calcium chloride, 30 mL dry dichloromethane, (53,52 mmol, 8,49, 1.25 eq) aluminum chloride and (3.81 ml, 1.05 eq) acetyl chloride were stirred in ice/salt bath. Using a syringe methyl homovanillate **1 (**20 g, 101.94 mmol, 1eq) dissolved in 175 mL dichloromethane was added while cooling in an ice/salt bath to keep the inner temperature below 20 °C. The reaction was allowed to stir at room temperature for 24h monitored with TLC. The yellow solution obtained was quenched with 100 mL of ice and 35 mL 1M HCl solution. The organic layer was then extracted with dichloromethane (3x50 mL) and washed with 1M NaOH solution (3x50 mL). The organic solution was dried over $MgSO_4$ and concentrated to obtain a yellow oil which was purified by column chromatography in 1:1 ethyl acetate-hexane mixture to obtain **2** as colorless solid (11.66 g, 48,94 mmol, 48%).

Alternatively, the classical Schotten-Baumann reaction with pyridine as HCl scavenger can be applied. The yields of raw product are even higher; however, the purification seems to be more complicated.

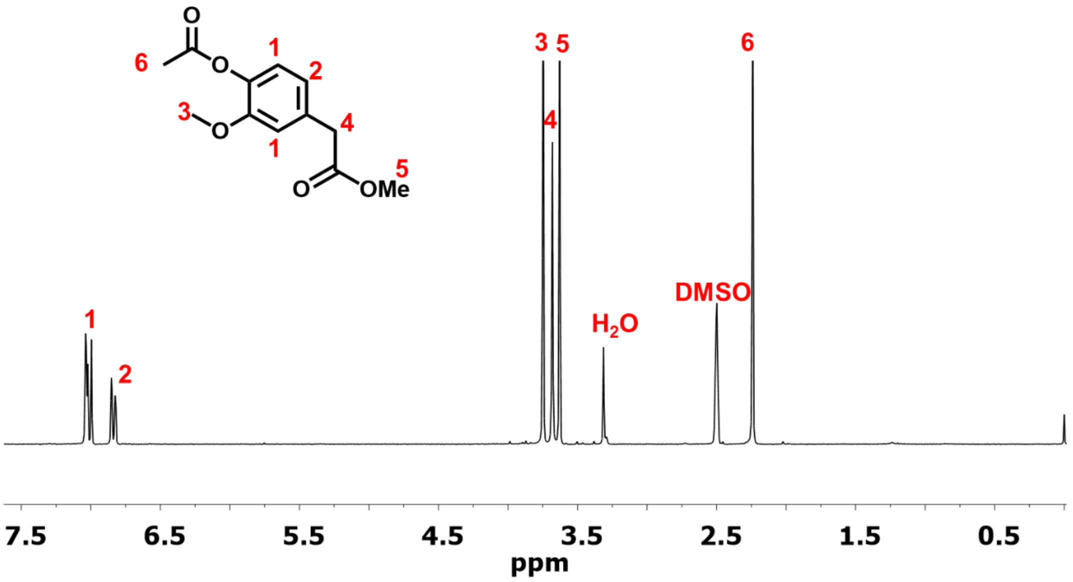

Figure S 3: ^{1}H-NMR (DMSO-d, 400MHz): δ [ppm] =7.022 (m, 2H, 1), 6.852 (d, 1H, 2), 3.747 (s,3H, 3), 3.681 (s, 2H, 4), 3.628 (s, 3H, 5), 2.24 (s, 3H, 5)

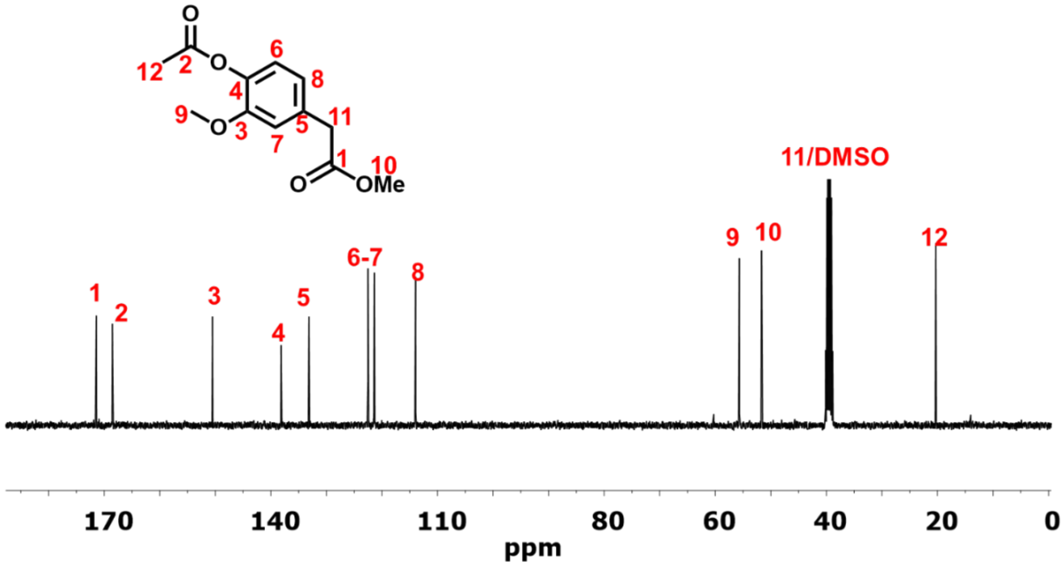

Figure S 4: ***^{13}C-NMR (DMSO-d, 400MHz):*** *δ [ppm] = 171.74 (1), 168.53 (2), 150.52 (3), 138.16 (4), 133.14 (5), 122.50 (6), 121.38 (7), 113.94 (8), 55.67 (9), 51.67 (10), 38.87 (11), 20.32 (12)*

1.3 Methyl 2-(4-acetoxy-2-formyl-5-methoxyphenyl)acetate (**3**):[3] [4]

Dichloromethyl methyl ether (15,93 mL, 146.91 mmol, 3.5 eq) was added to a solution of ester **2** (10 g, 41.97 mmol, 1 eq.) in dry 10 mL dichloromethane while stirring. Subsequently, $AlCl_3$ (22,39 g, 167.90 mmol, 4eq.) was added in several portions at 0 °C. The resulting solution was stirred for an additional 2 h at 0 °C and then for 24 h at rt. The reaction mixture was quenched by pouring into ice and then extracted with dichloromethane. The combined organic layer was washed with 5% potassium hydroxide solution, dried over sodium sulfate and concentrated in vacuo to give a cream white solid **3** (2.57 g, 9.66 mmol, 23%). The product was used without further purification

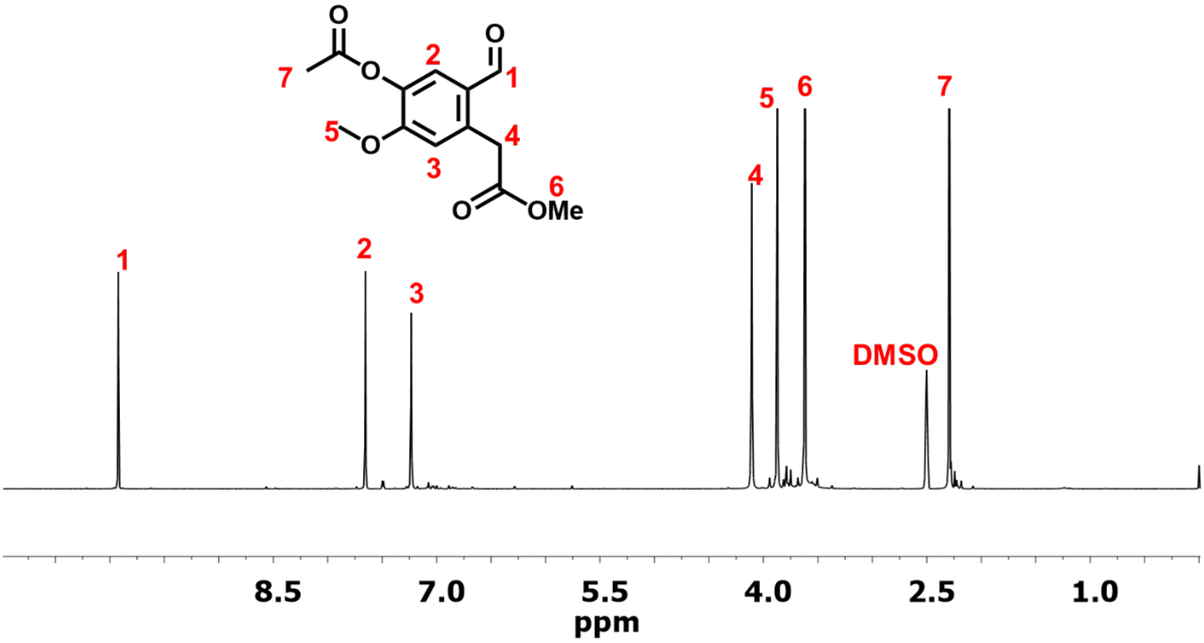

Figure S 5: ^{1}H-NMR (DMSO-d, 400MHz): δ [ppm] = 9.90 (s, 1H, 1), 7.65 (s, 1H, 2), 7.22 (s, 1H, 3), 4.11 (s, 2H, 4), 3.87(s, 3H, 5), 3.61 (s, 3H, 6), 2.29 (s, 3H, 7)

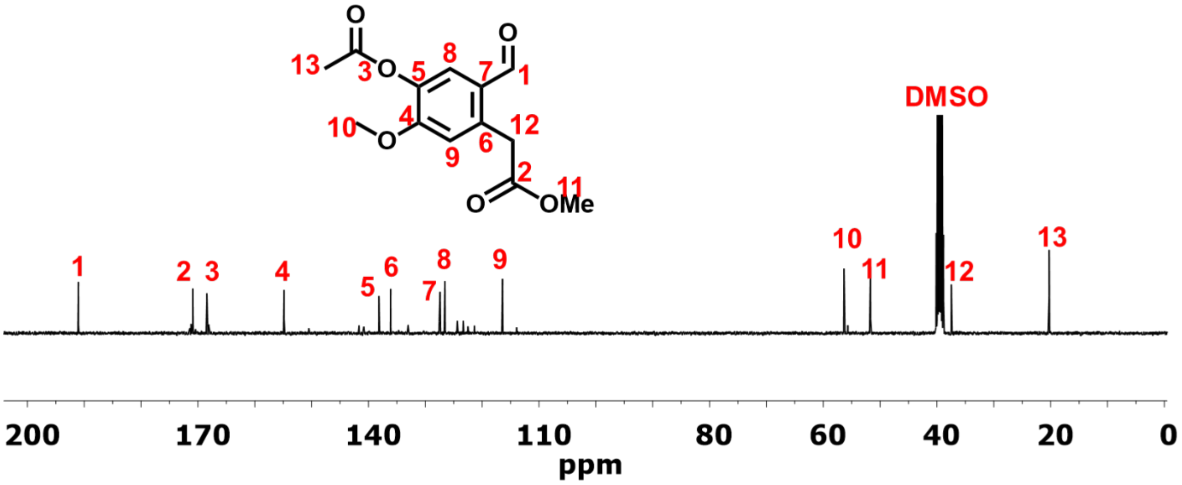

*Figure S 6: **^{13}C-NMR (DMSO-d, 400MHz):** δ [ppm] = 191.04 (1), 171.03 (2), 168.50 (3), 154.85 (4), 138.16 (5), 136.10 (6), 127.41 (7), 126.55 (8), 116.76 (9), 56.35 (10), 51.71(11), 37.47 (12), 20.32 (13)*

1.4 Methyl 2-(4-acetoxy-5-methoxy-2-((2-phenylacetamido)methyl)phenyl)acetate (**4**):[5]

Aldehyde **3** (2.5 g, 9.39 mmol, 1 eq) and phenylacetamide (1.65 g, 12.21 mmol, 1.1 eq) were dissolved in 17 mL anhydrous acetonitrile and trifluoroacetic acid (3.60 mL, 46.95 mmol, 5 eq) and triethylsilane (9 mL, 56.34 mmol, 6 eq) were added through a septum. The reaction was stirred at 50 °C until complete reaction (24 h, monitored by NMR). The reaction mixture was then concentrated in vacuo, diluted with chloroform, and washed with H_2O and NaOH solution. Further purification by column chromatography with a mixture of ethylacetate (1% acetic acid): hexane (2:1) was conducted to get a brown solid **4** (2.55 g, 21.97 mmol, 39%)

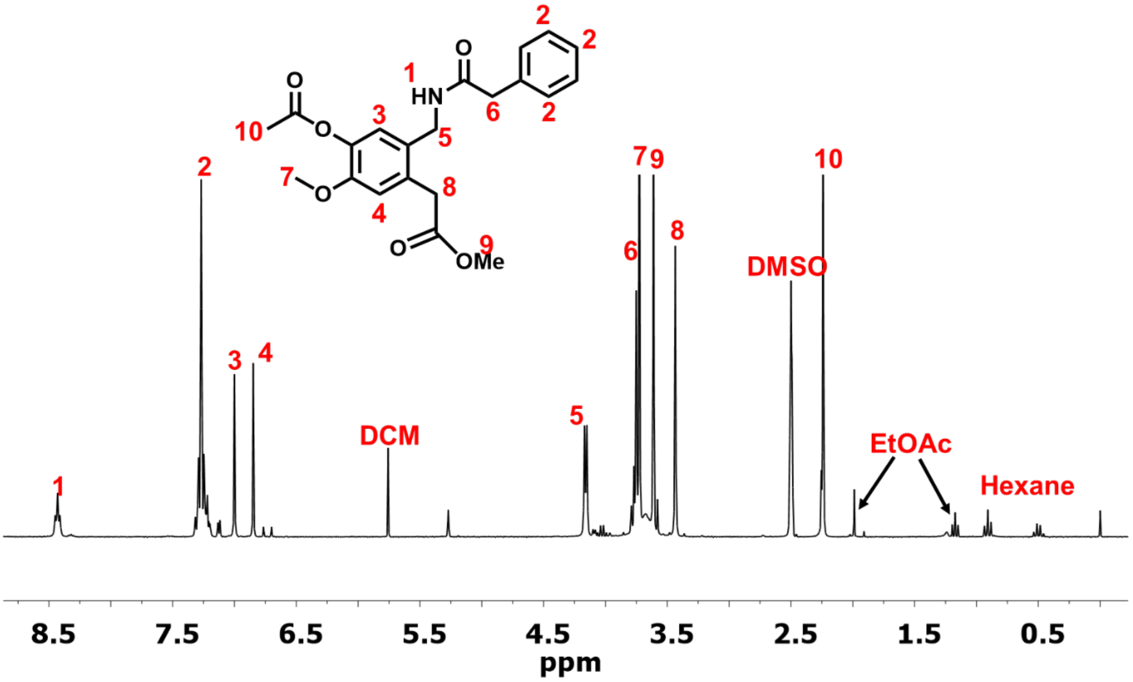

Figure S 7: ^{1}H-NMR (DMSO-d, 400MHz): δ [ppm]= 8.39 (s, 1H, 1), 7.26 (m, 5H, 2), 7.00 (s, 1H, 3), 6,85 (s, 1H, 4), 4.16 (d, 2H, 5), 3.75 (s, 2H, 6), 3.61 (s, 3H, 7), 3.55 (s, 3H, 9), 3.44 (s, 2H, 8), 2.24 (s, 3H, 10)

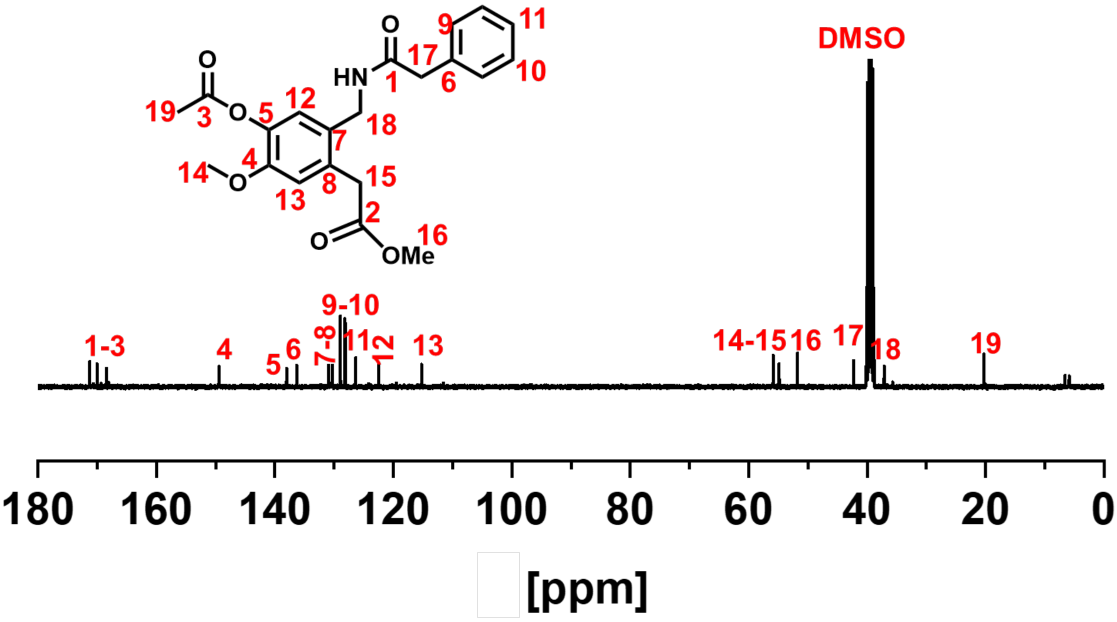

*Figure S 8: **^{13}C-NMR (DMSO-d, 400MHz):** δ [ppm] = 171.27 (1), 170.58 (2), 168.53 (3), 149.51 (4), 138.02 (5), 131.33 (7), 130.95 (8), 129.75 (9, 2C), 128.94 (10, 2C), 128.15 (11), 122.33 (12), 117.5 (13), 55.84 (14), 54.86 (15), 51.76 (16), 42.64 (17), 37.15 (18), 20.32 (19)*

1.5 2-(4-Hydroxy-5-methoxy-2-((2-phenylacetamido)methyl)phenyl)acetic acid (**5**):[6]

To the ester **4** (2 g, 4.80 mmol, 1eq) in 10 mL methanol/tetrahydrofuran (1:1), 30 mL 2 M aqueous lithium hydroxide was added, and the mixture was stirred at 50 °C for 24h. The reaction mixture was then concentrated in vacuo, acidified with 2 M aqueous hydrochloric acid to pH = 3, and then extracted with ethyl acetate (2 x 75 mL). The combined organic phases were washed with brine, dried over magnesium sulfate, and concentrated in vacuo to obtain a brown oil **5** (916 mg, 2.78 mmol, 58 % yield)

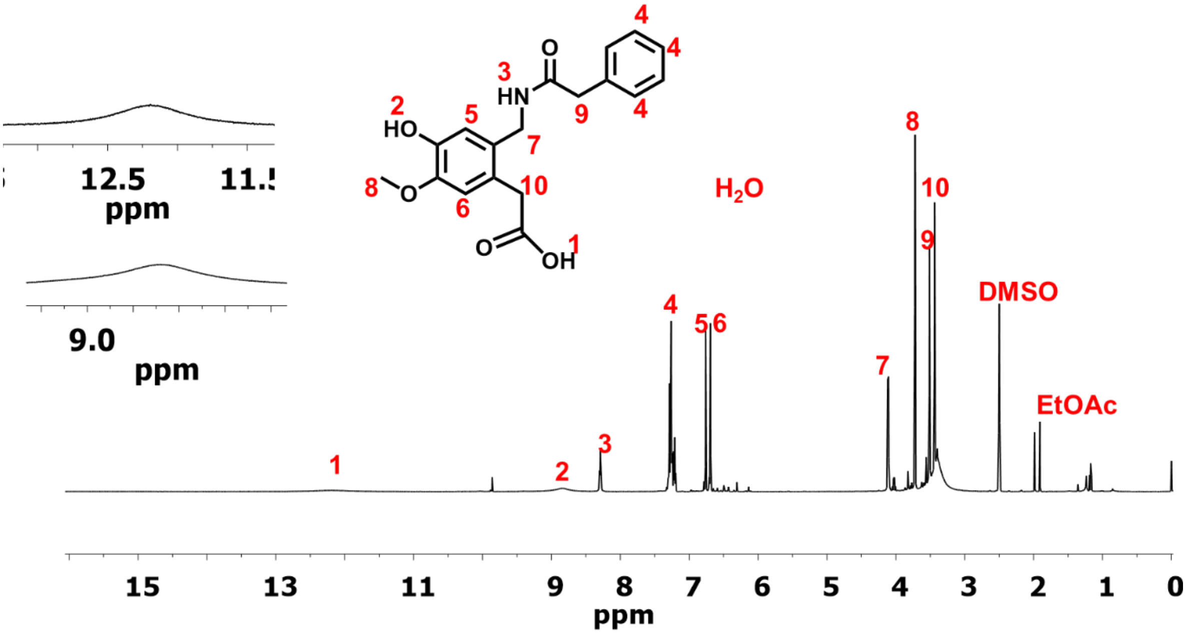

Figure S 9: ^{1}H-NMR (DMSO-d, 500MHz): δ [ppm]= 11.95 (s, 1H, 1), 8.91 (s, 1H, 2), 8.32 (t, 1H, 3), 7,25 (m, 5H, 4), 6.76 (s, 1H, 5), 6.70 (s, 1H, 6), 4.09 (d, 2H,7), 3.72 (s, 3H, 8), 3.62 (s, 2H, 9), 3.58 (s, 2H, 10)

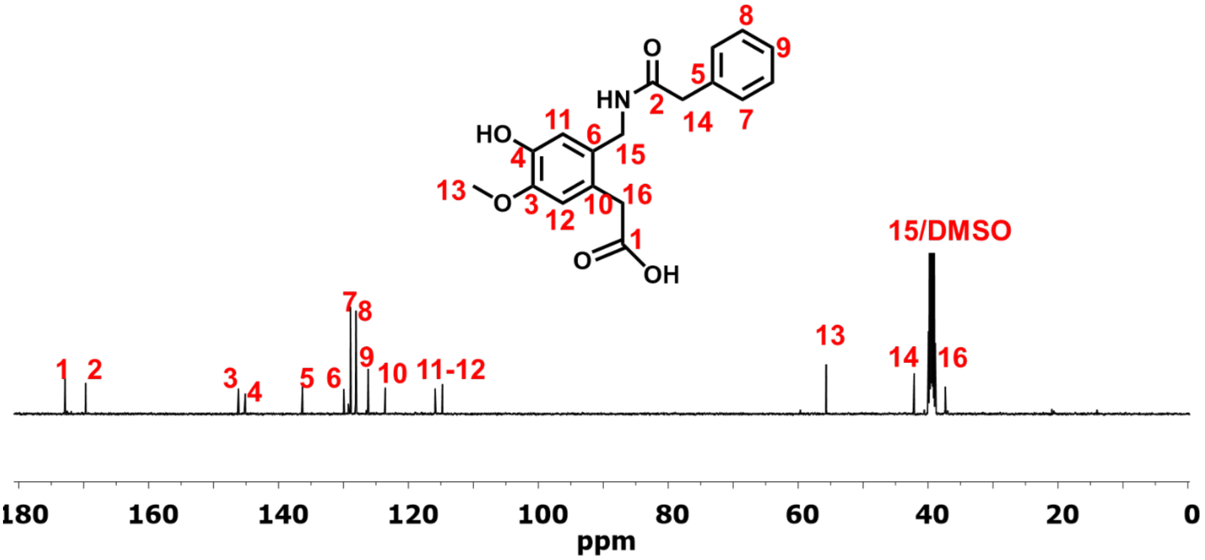

*Figure S 10: ^{13}**C-NMR (DMSO-d, 500MHz):** δ [ppm] = 171.86 (s, 1), 169.75 (s, 2),146.34 (s, 3), arom), 145.39 (s, 4), 136.38 (s, 5), 130.09 (s, 6), 129 (s, 2C, 7), 128.14 (s, 2C, 8), 126.29 (s, 9), 122.94 (s, 10), 116.6 (s, 11), 114.85 (s, 12), 55.76 (s, 13), 42.53 (14), 40.1 (s, 15), 36.91 (s, 16)*

1.6 1-Cyclopropyl-6-floro-7-(4-(2-(4-hydroxy-5-methoxy-2-((2-phenylcetamido)methyl)phenyl)acetyl)piperazin-1-yl)-1,4dihydroqinoline-3-carboxylic acid (**6**):[7]

In a Schlenk line flask previously degassed with N_2, ciprofloxacin 414.41 mg (1.25mmol, 1.2 eq) and N, N-diisopropylethylamine 1.26 mL (5.3 mmol. 5.8 eq, 0.742 g/mL, based on cipro) were mixed in 5 mL of dry dichloromethane/THF (4:1) and trimethylsilyl chloride (0.423 mL, 3.34 mmol, 3.2 eq). Separately, the linker system **5** (343.26 mg, 1.04 mmol, 1eq), PyAOP 706.43 mg (1.35 mmol, 1.3 eq) and N, N-diisopropylethylamine 0.49 mL (2.81 mmol, 2.7 eq base on linker) were dissolved in 5 mL of dry dichloromethane/THF (4:1). The two solutions were combined and stirred overnight at 23°C, under N_2 atmosphere. 80 mL H_2O was added and then extracted with 3x80 mL dichloromethane. The combined organic layers were washed with 80 mL H_2O, 10 % w/w citric acid (3 x 50 mL), saturated aqueous $NaHCO_3$ solution (3 x 50 mL), brine (2 x 50 mL), and finally, 4x50 mL H_2O. It was dried over $MgSO_3$ and concentrated under reduced pressure. A rust brown solid **6** was obtained (618 mg, 098 mmol, 94% yield)

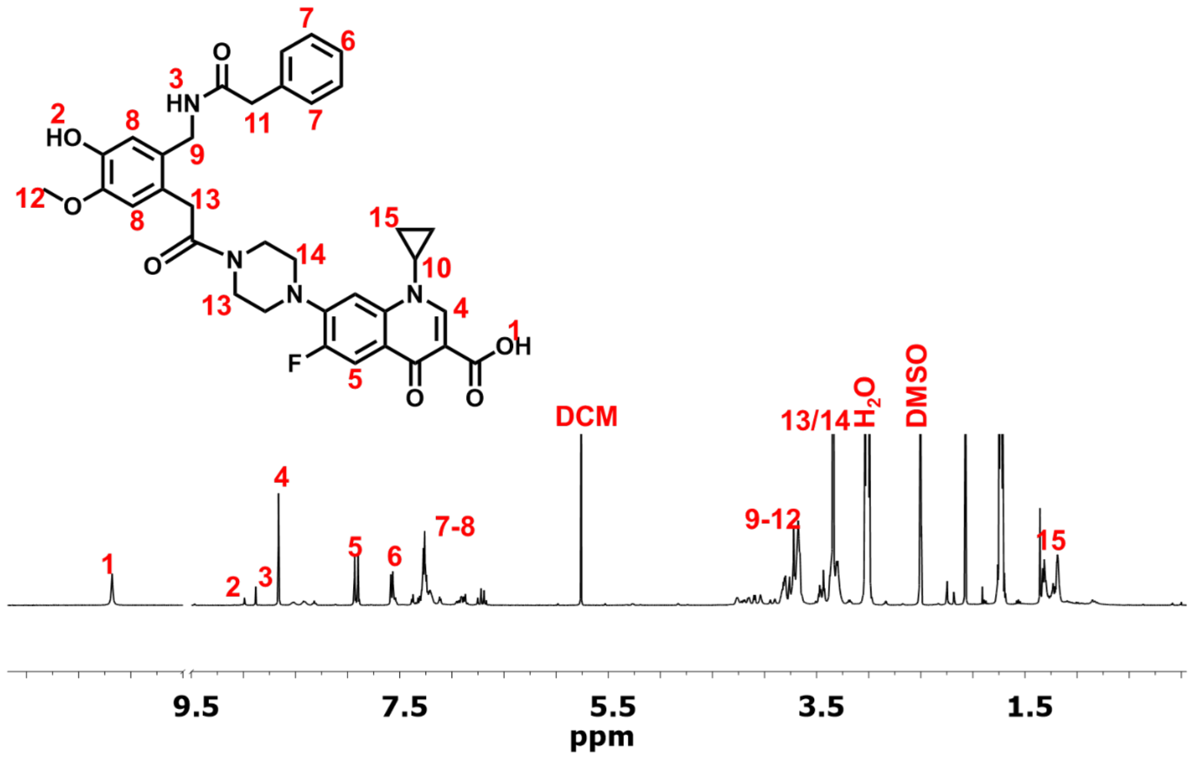

*Figure S 11: ^{1}**H NMR (400 MHz, DMSO-d):** δ [ppm] = 15.15 (s, 1H, 1), 8.99 (s, 1H, 2), 8.88 (d, 1H, 3), 8.63 (s, 1H, 4), 7.87 (1H, 5), 7.54 (dd, 1H, 6), 7.45 – 6.77 (m, 6H,7-8), 429 – 3.60 (m, 10H, 9-12), 3.52 (m, 4H, 13), 3.10 (m, 4H, 14), 1.37 – 1.12 (m, 4H, 15).*

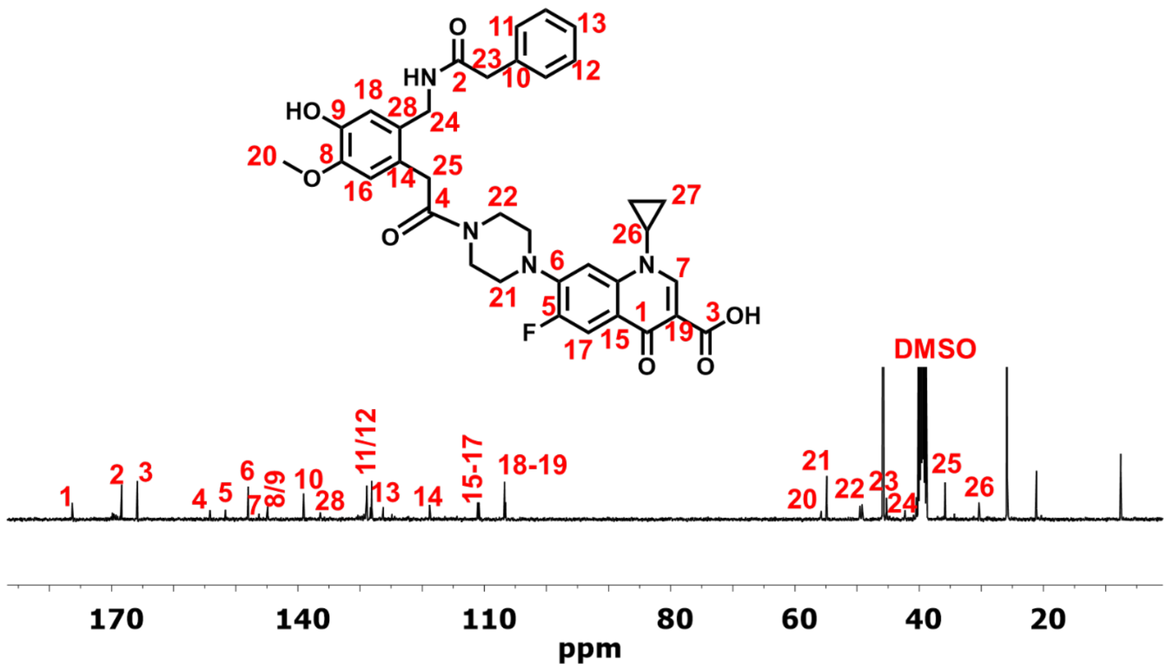

*Figure S 12: **^{13}C NMR (400 MHz, DMSO):** δ [ppm] = 176.29 (1), 168.460 (2), 165.76 (3), 154.16 (4), 151.68 (5), 148.16 (6), 146.34 (7), 144.96 (2C, 8/9), 139.37 (10), 136.36 (28), 128.62 (4C, 11-12), 126.44 (13), 118.78 (14), 111.23 (3C, 15-17), 107.14 (2C, 18-19), 56.22 (20), 55.2 (2C, 21), 49.55 (2C, 22), 45.28 (23), 42.29 (24), 35.84 (25), 30.25 (26), 7.91 (2C, 27).*

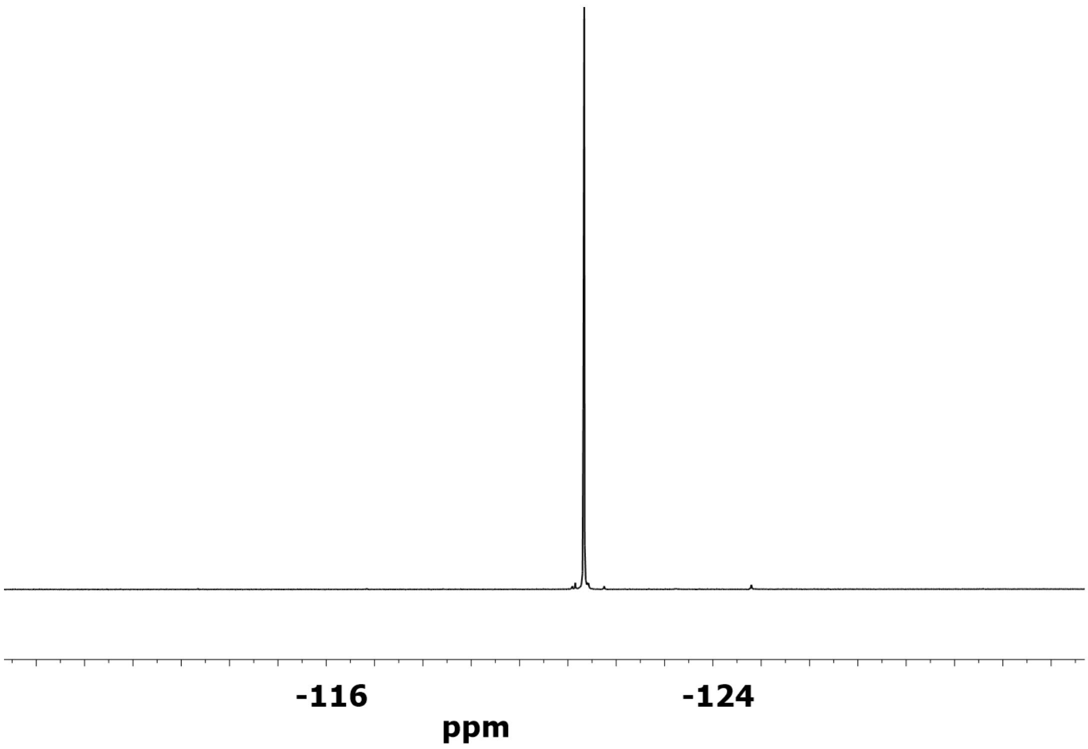

Figure S 13: ^{19}F NMR (400 MHz, DMSO-d): δ [ppm] = -121.3

1.7 2-(Bromomethyl)furan **7**

In a flask protected from light with aluminum foil (0.177 mL, 2.04 mmol) furfuryl alcohol was given into 1 mL dry diethyl ether and cooled to 0 °C. To this solution, phosphorus tribromide (0.232 mL, 2.45 mmol) was then added in portions while stirring. The mixture was stirred for 30 min at room temperature, and then transferred to a clean Erlenmeyer flask and 10 mL of a 40% aqueous sodium hydroxide solution was added at 0 °C. The organic phase was collected and dried over $MgSO_4$. Due to the high instability of 2-(bromomethyl) furan, the solvent was evaporated using a rotary evaporator at 25-30 °C by gradually reducing the pressure. A brown product **7** was obtained and used immediately for the next synthesis (262,59 mg, 1,63 mmol, 80 % yield)

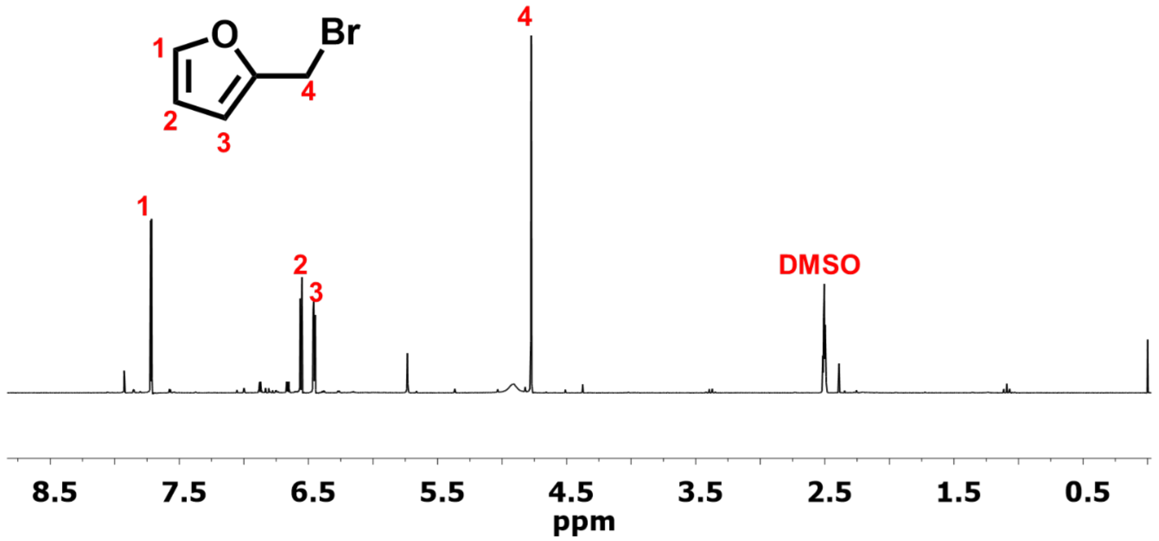

Figure S 14: ***^{1}H NMR (300 MHz, DMSO):*** *δ [ppm] = 7.72 (d, 1H, 1), 6.55 (dd, 1H, 2), 6.45 (d, 1H, 3), 4.78 (s, 2H, 4).*

1.8 1-Cyclopropyl-6-fluoro-7-(4-(2-(4-(furan-2-ylmethoxy)-5-methoxy-2-((2-phenylacetamido)methyl)phenyl)acetyl)piperazin-1-yl)-1,4-dihydroquinoline-3-carboxylic acid (**8**):[8]

In round bottom flask containing **6** (200 mg, 318,12 µmol, 1eq.), 2-(bromomethyl) furan (56.34 mg, 349.93 µmol, 1.1 eq) and K_2CO_3 (131,90 mg, 954.35 µmol, 3 eq) were dissolved in 3 mL dry DMF. The reaction was stirred at 70 °C for 24h. The solution was concentrated under reduced pressure and then rediluted with dichloromethane. The organic phase was washed with water (3x50 mL), dried over $MgSO_4$ and then evaporated to give a brown colored liquid **8** (182.63 mg, 257.68 mmol, 81%)

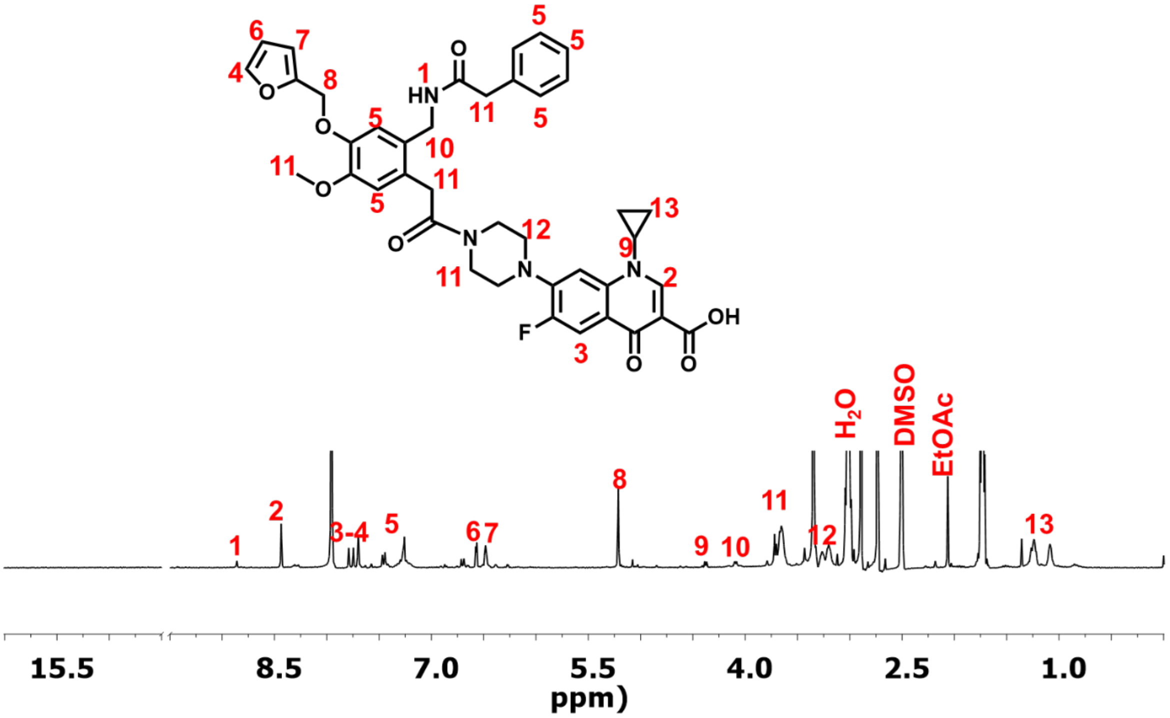

*Figure S 15: ^{1}**H NMR (300 MHz, DMSO):** δ [ppm] = 8.86 (d, 1H, 1), 8.44 (s, 1H, 2), 7.96 (s,1H, 3), 7.77 (d, 1H, 4), 7.44-7.12 (m, arom, 7H, 5), 6.57 (d, 1H, 6), 6.48 (dd, 1H, 7), 5.19 (s, 2H, 8), 4.37 (t, 1H, 9), 4.12 (d, 2H, 10), 3.77-3.54 (d, 11H, 11), 3.23 (d, 4H, 12), 1.19 (m, 4H, 13)*

1.9 Purification of chitosan:[9]

In a 250 mL round bottom flask equipped with a magnetic stirrer, 5 g commercial chitosan was suspended in 63.25 mL of 1 M NaOH solution and heated in to 70 °C under reflux condition for 2h. The suspended chitosan was filtered and washed with 100 mL distilled water (x4). The chitosan was then dissolved in 500 mL of 1 % acetic acid solution by stirring overnight. The solution was then filtered to remove any undissolved material. The solution was further purified by dialysis (MWCO= 14 kDa) against 0.1 M NaCl solution for 2 days and distilled water for 1 day. The chitosan was then freeze-dried.

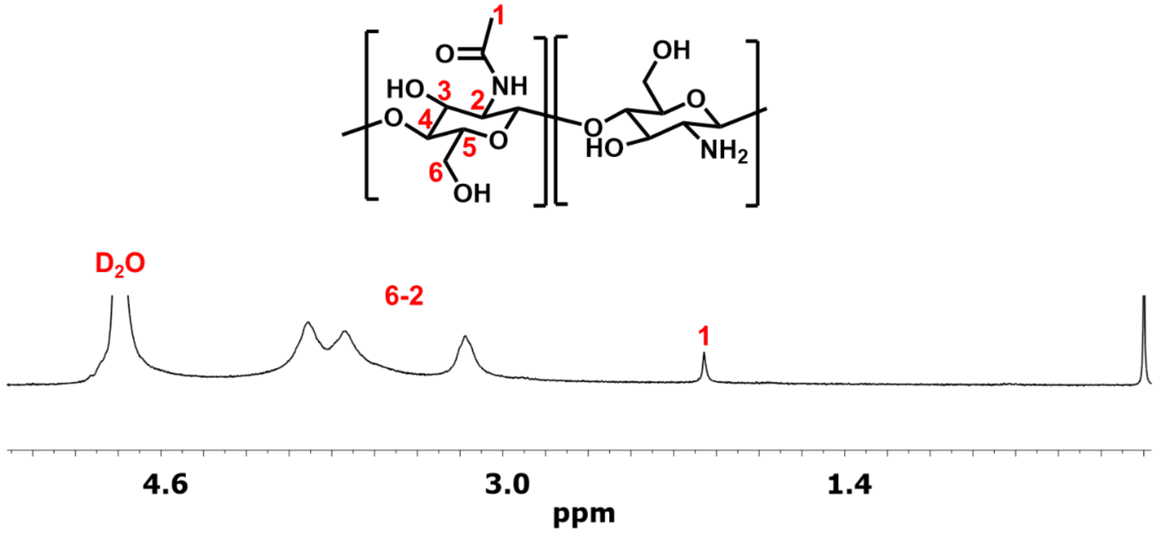

Figure S 16: ***^1H NMR (300 MHz, D_2O) δ [ppm]*** *= 4.17-2.80 (6H, 2-6), 2.02 (3H, 1)*

1.10 Modification of chitosan with 3-maleimidopropanoic acid (9):[10]

In a 500 mL round bottom flask containing a magnetic stirrer, pure chitosan (CS) (1 g, 4.93 mmol, 1eq) and 1-hydroxybenzotriazol (HOBt) (199.68 mg, 2 eq based on maleimide) were dissolved/suspended in 60 mL of Milli-Q water and stirred overnight. To the CS solution, 200 mL of dimethyl sulfoxide (DMSO) and 3-maleimidopropionic acid (124.97 mg, 0.74 mmol, 0.15 eq; base on CS) were added. Subsequently, 1-ethyl-3- (3-dimethyl aminopropyl-carbodiimide) (EDC) (169.98 m, 1.2 eq based on maleimide) was added and a starting pH of 5.80 was recorded. The reaction was stirred for 24h at rt. The reaction mixture was dialyzed against a solution of 10^{-2} M HCl and 1 wt% NaCl for 2 days, followed by dialysis with a 10^{-2} M HCl solution for 1 day. The dialysis media were exchanged three times daily. Finally, the sample was freeze-dried.

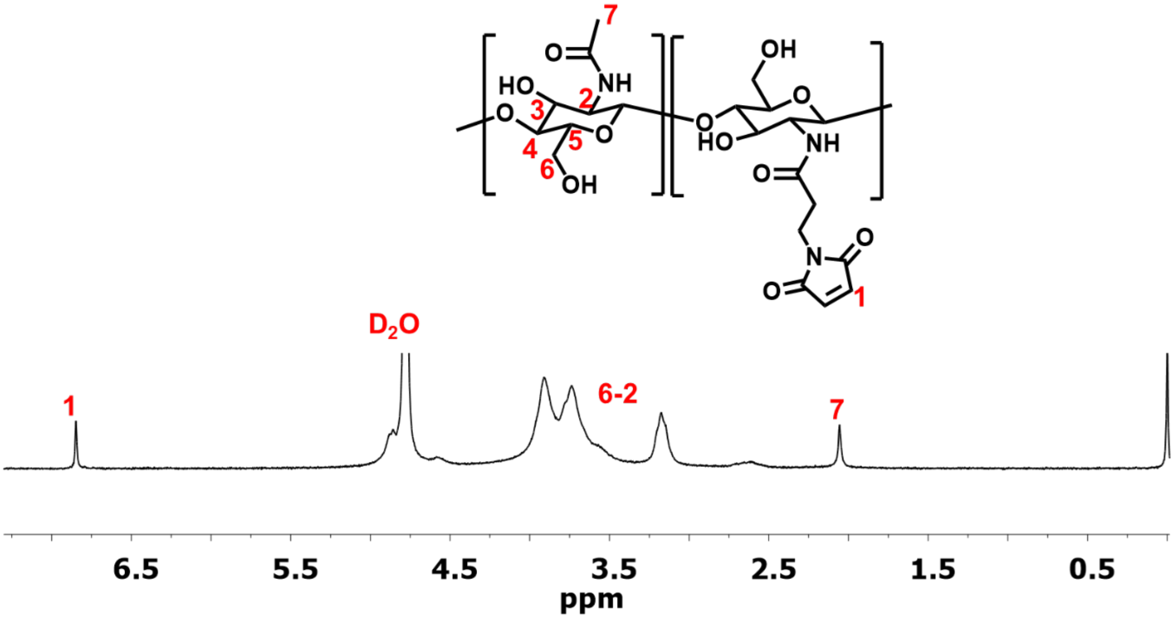

*Figure S 17: ^{1}**H NMR (300 MHz, D_2O) δ [ppm]** = 6.85 (s,2H,1), 4.17-2.80 (6H, 2-6), 2.02 (3H, 7)*

1.11 Chitosan-drug delivery system (10):[11]

In a round bottom flask charged with a magnetic stirrer functionalized maleimide functionalized chitosan (CS-MI) **9** (300 mg, 1.72 mmol) was dissolved in 2 % acetic acid solution (15 mL). The furan modified linker **8** (181.68 mg, 0.25 mmol, 0.17 eq based on functionalized units only) was dissolved separately in DMSO (5 mL) and the two solutions were mixed and allowed to stir for 24h at 65 °C. Purification was conducted by dialysis against 1 M HCl acid solution (MWCO = 12-14 kDa) for 24h (3X). The sample was freeze dried to remove all solvents to give 150.2 mg of **10.**

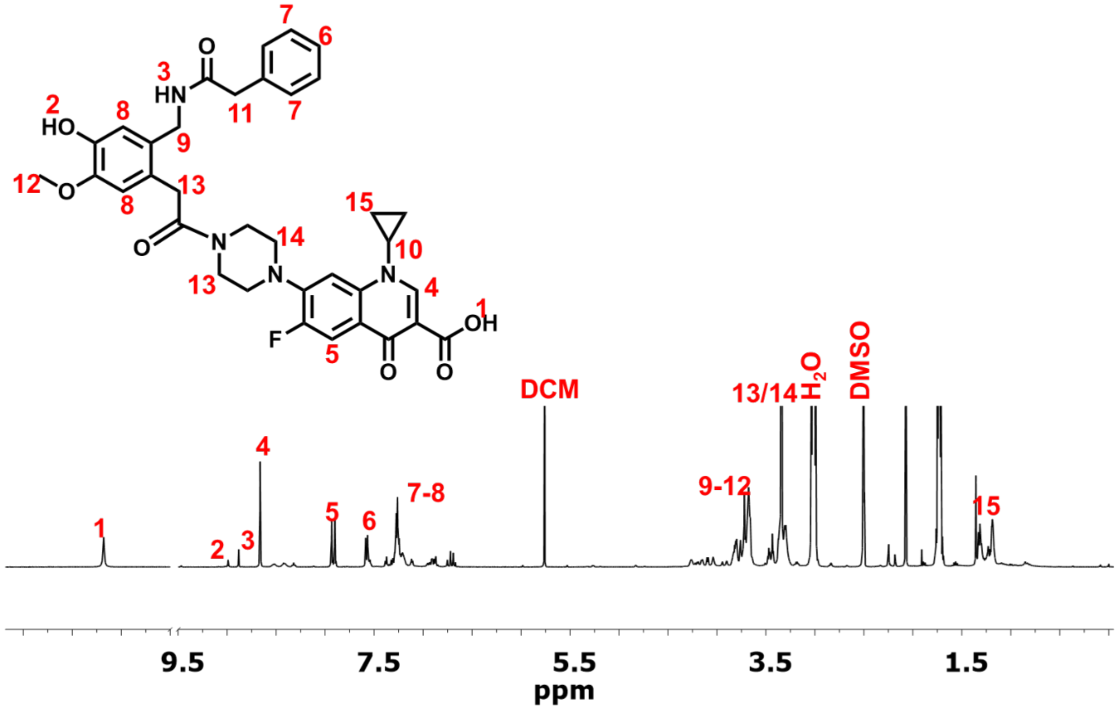

*Figure S 18: ^{1}**H NMR (400 MHz, DMSO-d):** δ [ppm] = 15.15 (s, 1H, 1), 8.99 (s, 1H, 2), 8.88 (d, 1H, 3), 8.63 (s, 1H, 4), 7.87 (1H, 5), 7.54 (dd, 1H, 6), 7.45 – 6.77 (m, 6H,7-8), 429 – 3.60 (m, 10H, 9-12), 3.52 (m, 4H, 13), 3.10 (m, 4H, 14), 1.37 – 1.12 (m, 4H, 15).*

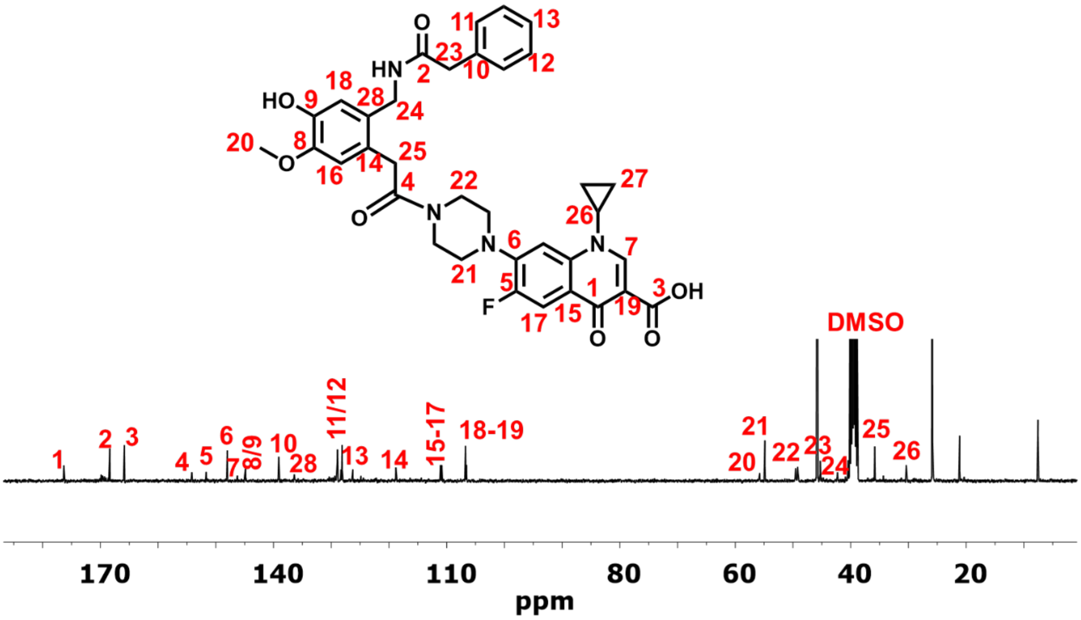

*Figure S 19: ^{13}**C NMR (400 MHz, DMSO):** δ [ppm] = 176.29 (1), 168.460 (2), 165.76 (3), 154.16 (4), 151.68 (5), 148.16 (6), 146.34 (7), 144.96 (2C, 8/9), 139.37 (10), 136.36 (28), 128.62 (4C, 11-12), 126.44 (13), 118.78 (14), 111.23 (3C, 15-17), 107.14 (2C, 18-19), 56.22 (20), 55.2 (2C, 21), 49.55 (2C, 22), 45.28 (23), 42.29 (24), 35.84 (25), 30.25 (26), 7.91 (2C, 27).*

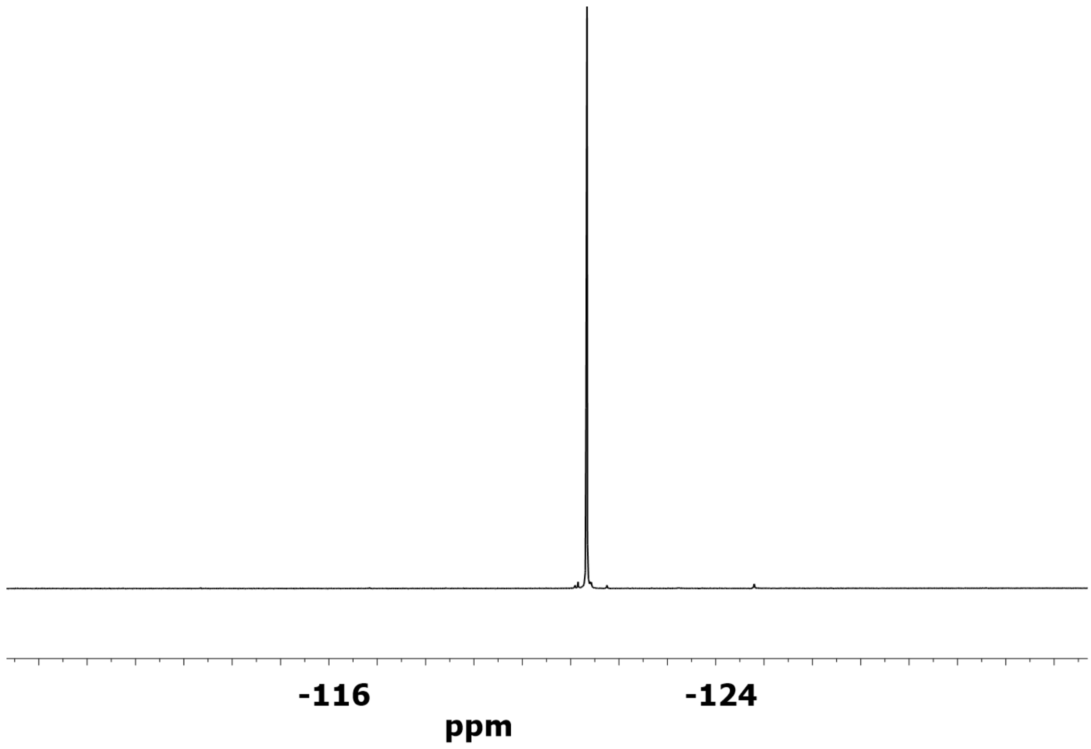

Figure S 20: ^{19}F NMR (500 MHz, DMSO-d): δ [ppm] = -119.23, 120.5 and 120.45 (See Figure 3)

1.12 Purification of alginate:[12]

The brown sodium alginate was further purified by dissolving 1 wt% sodium alginate in water under vigorous stirring. The solution was extracted three times with chloroform/butanol (4:1, v/v), and the mixture was centrifuged at 3000 rpm to remove the denatured protein and chloroform/butanol phase. Activated charcoal was then added to the solution and filtered off. The solution was precipitated in absolute ethanol, and the precipitate was dissolved in water and filtered through 0.45 µm and 0.22 µm membrane filters. Finally, the filtrate was freeze-dried to obtain a white solid.

1.13 Formation of coating through dip-coating process and stability of coating

100 mg of **10** were dissolved in 25 mL of 2% acetic acid/DMSO solution (16 mL:9 mL). The pH of the solution was 2.37. Another solution of 100 mg of purified sodium alginate was prepared in distilled water, resulting in a pH of 6.72. For preparation of the multilayers, Ti-plates were polished with 320, 1000 silicon carbide sandpapers and with colloidal silica (type MasterMet 50 nm and MasterMed 20 nm) polishing solutions for varied times. Polishing materials were purchased from Buehler (Lake Bluff, IL, USA). The plates were washed with different solvents (chloroform, acetone, ethanol and deionized water) and air plasma cleaned for 10 min at 75 W (Diener electronic GmbH, Nagold, Germany). A dip robot (Riegler & Kirstein, Berlin, Germany) was used for preparation of the multilayer system. For a 10 layered film system, the coating was done according to Min et all.[13] The coating where formed at room temperature by alternate dipping in to a solution of the polycation (chitosan-bound linker), followed by consecutive rinsing steps in distilled water and drying under N_2 stream and then dipping into the polyanion solution (sodium alginate) solution followed by same rinsing and drying cycles. This process was repeated ten times. The dry layer thickness was measured by laser ellipsometry. An average for 3 different plates was considered for the layer thickness.

Furthermore, the stability studies of coating on Ti-plate were carried out by incubation of the coated Ti-plates in phosphate buffered saline. This was done at pH of 7.4 and at 37 °C. The coated Ti-plates were collected after 1 day, washed in distilled water, dried under N_2 atmosphere and the change in dried

layer thickness was measured in ellipsometry. The same procedure was repeated after 2days and after 14 days of incubation. After every measurement, the Ti-plates were incubated with a fresh solution to ensure an effective stability test.

1.14 Enzyme triggered release of ciprofloxacin from coatings

Release of ciprofloxacin was investigated by incubating the coated Ti-Plates (non polished) in 2 mL of penicillin-G-amidase (PGA, Penicillin Amidase from Escherichia coli, Sigma Aldrich, Taufkirchen, Germany) at 4 mg/mL in 0.01 M sodium phosphate buffered saline (PBS, pH 7.4) for 20 h at 37 °C. Subsequently, the buffer solution was diafiltrated (Amicon® Ultra Centrifugal Filter, 30 kDa MWCO) at 3000 rpm for 10 min to remove the enzyme. The filtrate was investigated by UHPLC-MS and MS-MS.

1.15 UHPLC-MS and MS/MS measurements

High-resolution accurate-mass (HRAM) measurements of the ciprofloxacin solutions were performed using a Vanish Horizon ultra-high-performance liquid chromatography (UHPLC) system from Thermo Fisher Scientific, comprising a VH-P10-A pump, a VH-A10-A autosampler and a VH-C10-A column oven (not temperature-controlled), coupled to an Exploris 120 HRMS Orbitrap instrument (Thermo Fisher Scientific, Waltham, USA). The MS method included the following settings: H-ESI spray voltage (positive) of 3,500 V; ion transfer tube temperature and vaporizer temperature of 325 °C and 350 °C, respectively. The sheath gas, aux gas and sweep gas were set to 50, 10 and 1 arb. units, respectively. Full scan HRAM data were recorded for the m/z range of 150–1000 at an Orbitrap resolution of 120,000. Alternating targeted MS/MS spectra for m/z 332.1405 (isolation window was m/z 1) were recorded at an Orbitrap resolution of 15,000 and a normalized HCD collision energy of 30%. Prior to each UHPLC-MS run, a one-point mass calibration was performed using the built in EASY-IC.

Chromatographic separation was achieved using a Raptor Biphenyl analytical column 150 mm × 2.1 mm, 2.7 µm (Restek, Bad Homburg, Germany), including a guard column of the same material (5 mm × 2.1 mm). Mobile phase A consisted of water containing 0.1% formic acid, and mobile phase B consisted of methanol containing 0.1% formic acid. The flow rate was 0.250 mL/min, with a chosen injection volume of 5 µL. The separation gradient was 5% B (0–2 min), followed by a gradient to 100% B from 2 to 17 min. These conditions were then maintained at 100% B (17–21 min), before changing back to 5% B (21–22 min), followed by a final re-equilibration step at 5% B (22–32 min).

Data acquisition and evaluation were performed using Thermo Scientific Xcalibur Version 4.6.67.17, Thermo FreeStyle 1.8 SP2, and Thermo Xcalibur Qual Browser 4.4.67.17. Reconstructed ion chromatograms (RICs) for ciprofloxacin were generated using m/z 332.1405 and a mass tolerance of ±2.5 ppm, which corresponds to the protonated pseudo molecular ion of ciprofloxacin, $[C_{17}H_{18}FN_3O_3+H]^+$.

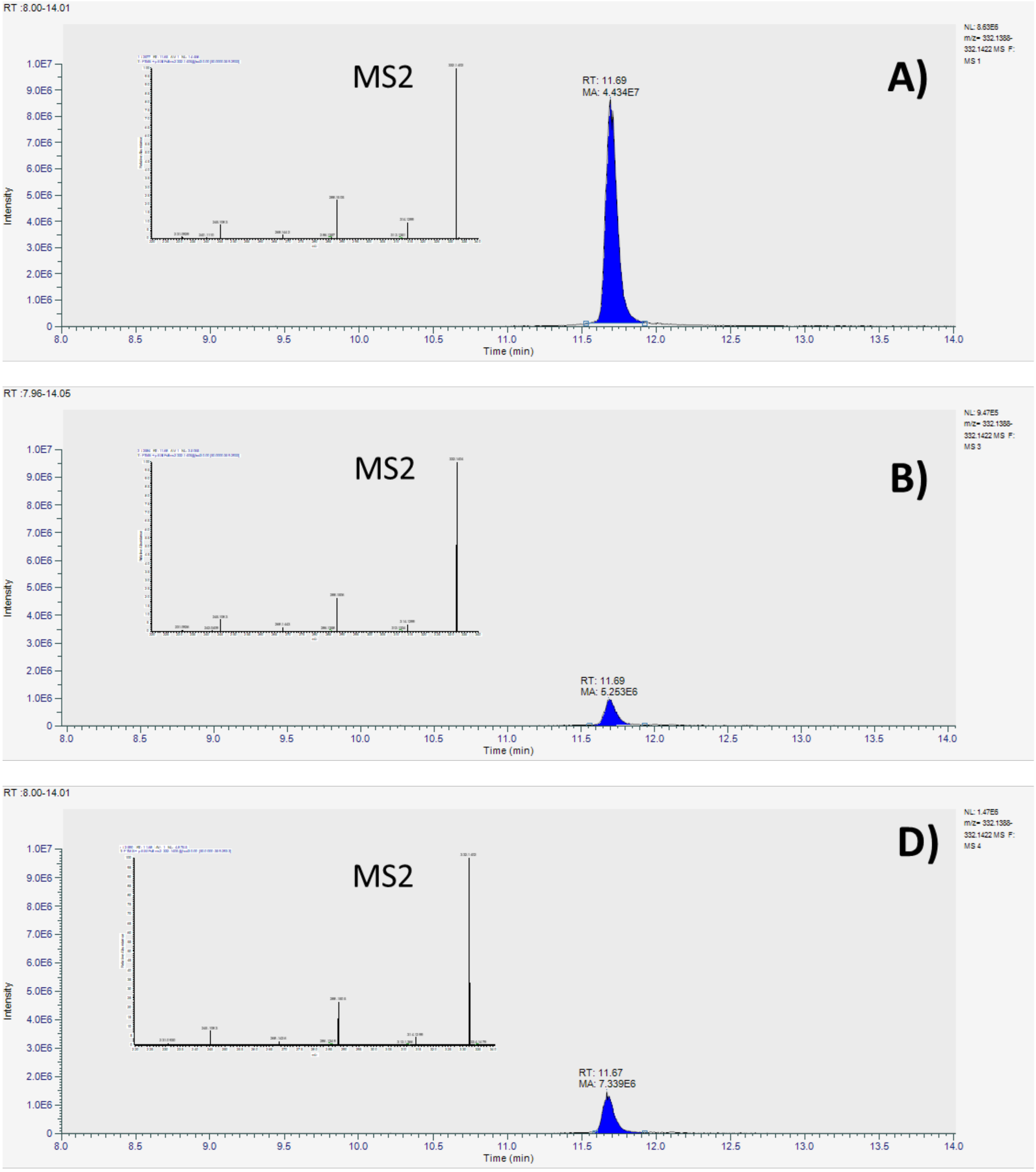

Figure S 21:Comparison of UHPLC-MS RICs for the enzyme triggered release of ciprofloxacin from titanium plates coated by LbL with 10 alternating layers of ***10*** *and alginate using m/z 332.1405 and a mass tolerance of ±2.5 ppm. This corresponds to the protonated pseudo molecular ion of ciprofloxacin, $[C_{17}H_{18}FN_3O_3+H]^+$. To prove authenticity, the corresponding MS^2 spectra for each peak are shown. A) Addition of active PGA, B) No PGA added, and C) Deactivated PGA added.*

1.16 Solubility of the linker-drug compound 6 and experimental groups

The linker-drug compound **6** was solubilized in 100 % dimethyl sulfoxide (DMSO, Sigma Aldrich, Taufkirchen, Germany) for 3 h under shaking conditions. This solution was further diluted using 96 % of 0.1 M sodium phosphate buffered saline (PBS, pH 7.4). To consider any effects caused by this solvent a mixture of 4% DMSO with 96 % PBS was used for all experimental groups. Two different volumes of compound (pure solution => 1:1; pre-diluted solution mixed with the same amount of

respective culture medium => 1:3) were considered. 10 mg/mL penicillin-G-amidase (PGA, Penicillin Amidase from Escherichia coli, Sigma Aldrich, Taufkirchen, Germany) was used for linker cleavage and ciprofloxacin release. The direct effect of PGA and of different concentrations of ciprofloxacin (1 to 16 µg/mL) was also analyzed separately.

1.17 Cytocompatibility testing of the linker-drug compound 6 using HGFs

To evaluate the cytocompatibility of the linker-drug compound **6** regarding HGFs, assays for membrane integrity and metabolic activity were performed as well morphologic changes were documented. Primary HGFs (1210412, Provitro AG, Berlin, Germany) were seeded in 100 µL of Dulbecco Modified Eagle Medium (P04-03591, Pan-Biotech, Aidenbach, Germany) supplemented with 10% FBS (Pan-Biotech, GmbH, Aidenbach, Germany) and 1 % v/v penicillin/streptomycin (A2212, Biochrom GmbH, Berlin Germany) at a density of $1x10^4$ cells/mL and cultured in a 96-well plate for 24 h at 37 ˚C in a humidified atmosphere with 5 % CO_2. After 24 h, cell culture medium was supplemented with 100 µL of the experimental groups. For the positive control 100 µL DMEM was added and for complete cell lysis in the negative control 1 % Triton-X 100 (Sigma-Aldrich Chemie GmbH, Taufkirchen, Germany) in DMEM. To consider the background effects of components on assays, the same mixtures were prepared in 96-plates without HGFs (background control plate). Both plates were incubated for 24 h in a humidified atmosphere.

After incubation, cell morphology was documented using a compound light microscope (Leica DMi1, Leica Microsystem GmbH, Mannheim, Germany). To measure the release of LDH, 100 µL of supernatants from each group were transferred to a new plate and mixed with the LDH working solution (11644793001, Roche Diagnostics, Germany) for 15 min. The reaction was stopped by adding 50 µL of 1 M HCl. The absorbance was measured using a spectrophotometer plate reader (Infinite 200 Pro, Tecan Group Ltd., Männedorf, Switzerland) at 490 nm (reference 690 nm) wavelength. To assess the cell metabolic activity, 20 µL of staining solution (Cell Titer-blue, Promega GmbH, Mannheim, Germany) was added to the remaining volume of cell culture medium (100 µl) and incubated for 3 h in a humidified atmosphere. The metabolic reduction of resazurin to fluorescent resorufin was measured at 560/590 nm using a spectrophotometer plate reader. In both cases the background values (without cells) were subtracted. The values for each group were converted into percentages using values of positive and negative control groups for normalization. All the experiments were performed in triplicates for each group.

1.18 Antibacterial activity of linker-drug compound **6** against *A. naeslundii*

To investigate the antibacterial activity of linker-drug compound **6**, the growth of *A. naeslundii* was evaluated using broth dilution assay and CFUs analysis. *A. naeslundii* (DSM 43013) obtained from the German Collection of Microorganisms and Cell cultures (DMSZ, Braunschweig, Germany), were stored in glycerol stocks. To obtain fresh colonies, bacteria were cultured for 48 h on fastidious anaerobe agar plates (FAA, Oxoid Limited, Wesel, Germany) containing 5 % sheep blood (Thermo Fisher Scientific, Darmstadt, Germany) under anaerobic conditions (80 % N_2, 10 % H_2, 10 % CO_2) at 37 °C. Fresh colonies were inoculated overnight in brain heart infusion (BHI, Oxoid Limited, Wesel, Germany) medium supplemented with 10 µg/mL vitamin K (Carl Roth GmbH & Co. KG, Karlsruhe, Germany) under anaerobic conditions. The bacterial cultures were further diluted in fresh BHI medium to obtain an OD of 0.001 at 600 nm. 100 µL of planktonic bacterial cultures were placed in 96-well plates and incubated for 1 h at 37°C under anaerobic conditions. Subsequently, bacteria cultures were supplemented with 100 µL of the experimental groups and further incubated for 24 h under anaerobic conditions. Absorbance was measured using a spectrophotometer plate reader at a wavelength of 600 nm and background control values (without bacteria) were subtracted. Bacterial activity was calculated for all groups as percentages of positive control (bacterial growth in BHI with vitamin K). For CFUs analysis, 100 µL of bacterial supernatant from each group were plated on FAA plates with 5 % sheep

blood and were incubated for 48 h at 37°C under anaerobic conditions before documented. All experiments were carried out in triplicates for each group.

1.19 Biocompatibility of chitosan-bound linker DDS coated on titanium plates

The biocompatibility of titanium plates was estimated by evaluating alterations in cell morphology, cell adhesion as well as membrane integrity of HGFs. This evaluation was performed under following conditions: uncoated titanium, uncoated titanium with PGA enzyme, titanium coated with chitosan-bound linker DDS, coated titanium with PGA enzyme, positive and negative control groups. HGFs cells were cultured on the samples at a $1x10^5$ cells/mL density in DMEM and were incubated for 24 h at 37 ˚C in a humidified atmosphere with 5 % CO_2. Subsequently, all samples were transferred to a new well plate containing fresh DMEM with or without PGA and were further incubated for 24 h.

To determine cell membrane integrity, the LDH activity in supernatants was measured using spectrophotometer as explained in 3.15. Alterations in cell morphology and cell adhesion were evaluated by fluorescence staining using confocal laser microscopy (CLSM, Leica TCS SP2, Leica Microsystems, Mannheim, Germany). Briefly, the adhered cells on samples were fixed using 4% paraformaldehyde for 20 min at room temperature. Cell membrane was permeabilize with 0.3 % Triton X-100 in PBS (Biochrom GmbH, Berlin, Germany) for 10 min at room temperature. The cells were stained at room temperature in the dark for 30 min. Actin filaments were labeled with Phalloidin green (Phalloidin-iFluor Reagent, abcam, Cambridge, UK) fluorescent dye, while a 4′,6-diamidino-2-phenylindol (DAPI) (Sigma-Aldrich Chemie GmbH, Taufkirchen, Germany) was used to mark cell nuclei. 2D images were obtained at 40 X at 10 different defined positions of samples. Imaris (Bitplane, Zurich, Switzerland) was used for the qualitative analyses.

1.20 Antibacterial activity of chitosan-bound linker DDS coated on titanium plates

To estimate the antibacterial activity of chitosan-bound linker DDS, the behavior of *A. naeslundii* biofilm was assessed by fluorescence staining using CLSM. Fresh colonies of *A. naeslundii* were cultured and were tested for uncoated and coated titanium plates under with or without enzymatic conditions. Samples were placed in BHI medium containing bacterial cultures (OD=0.01) and were incubated for 24 h at 37°C under anaerobic conditions. Subsequently, samples were transferred to a new well plate containing fresh BHI with or without PGA and were further incubated for 24 h.

For fluorescence staining, samples were washed twice with PBS solution and the biofilm was stained with Syto9 and propidium iodide dyes using the Live/Dead staining bacterial kit (Life Technologies, Darmstadt, Germany) for 30 min at a room temperature. Biofilm fixation was accomplished with 2.5% glutaraldehyde (Carl Roth, Karlsruhe, Germany) for 15 mins at 4 ˚C and the 3D images were obtained using CLSM. Syto-9 and propidium iodide were excited using 488 and 552 nm laser lines and images were obtained at 60-fold magnification with a 5 µm Z-step at 10 different sites. The images were evaluated for bacterial biofilm volume and cell membrane integrity using Imaris software. The Syto9 presented a 'green' signal suggesting an intact bacterial cell membrane (live) in contrast to propidium iodide that exhibited a 'red' signal highlighting cells with damaged bacterial membrane (dead). Colocalization staining was considered as 'dead' and were further subtracted from the total 'live' biofilm.

1.21 Statistical analysis

Data were analyzed using GraphPad Prism (La Jolla, CA, USA). The normality of data was assessed using the Shapiro Wilk and D'Agostino & Pearson tests. Differences between groups were compared using one- and two- way analysis of variance (ANOVA) for parametric data and the Kruskal-Wallis test for non-parametric data. Post hoc corrections were done using Tukey's and Dunn's tests. The significance level was set to α=0.05.

References

[1] T. Bozzini, G. Botta, M. Delfino, S. Onofri, R. Saladino, D. Amatore, R. Sgarbanti, L. Nencioni, A. T. Palamara, *Bioorg. Med. Chem.* 2013, *21*, 7699.

[2] G. D. Couch, P. J. Burke, R. J. Knox, C. J. Moody, *Tetrahedron* 2008, *64*, 2816.

[3] Joseph W. Meisel, Chunhua T. Hu, and Andrew D. Hamilton, *Org.Lett.* 2019, *21*, 7763.

[4] C. J. Stearman, M. Wilson, A. Padwa, *J.Org.Chem* 2009, *74*, 3491.

[5] Daniel DubS and Andrew A. Scholte, *Tetrahedron Lett.* 1999, *40*, 2295.

[6] Abdellah Ech-Chahad, Lahboub Bouyazzaa and Giovanni Appendino, *Nat. Prod. Commun.* 2006, *1*, 1147.

[7] T. Zheng, E. M. Nolan, *Bioorg. Med. Chem.* 2015, *25*, 4987.

[8] U. Grether, H. Waldmann, *Angew. Chem. Int. Ed.* 2000, *39*, 1629.

[9] Q. Gan, T. Wang, *Colloids Surf. B Biointerfaces* 2007, *59*, 24.

[10] M. Matsumoto, W. Udomsinprasert, P. Laengee, S. Honsawek, K. Patarakul, S. Chirachanchai, *Macromol. Rapid Commun.* 2016, *37*, 1618.

[11] Camille Chapelle, Baptiste Quienne, Céline Bonneaud, Ghislain David, Sylvain Caillol, *Carbohydr. Polym.* 2021, *253*, 117222.

[12] M. L. Torres, J. M. Fernandez, F. G. Dellatorre, A. M. Cortizo, T. G. Oberti, *Algal Res.* 2019, *40*, 101499.

[13] J. Min, R. D. Braatz, P. T. Hammond, *Biomaterials* 2014, *35*, 2507.

2
